# A Heterocatalytic Metal–Organic Framework to Stimulate Dispersal and Macrophage Combat with Infectious Biofilms

**DOI:** 10.1021/acsnano.2c09008

**Published:** 2023-01-24

**Authors:** Renfei Wu, Tianrong Yu, Sidi Liu, Rui Shi, Guimei Jiang, Yijin Ren, Henny C. van der Mei, Henk J. Busscher, Jian Liu

**Affiliations:** † Institute of Functional Nano and Soft Materials (FUNSOM), Jiangsu Key Laboratory for Carbon-Based Functional Materials and Devices, Joint International Research Laboratory of Carbon-Based Functional Materials and Devices, Soochow University, 199 Ren’ai Rd., Suzhou, Jiangsu215123, P. R. China; ‡ University of Groningen and University Medical Center Groningen, Department of Biomedical Engineering, Antonius Deusinglaan 1, 9713 AVGroningen, The Netherlands; § University of Groningen and University Medical Center of Groningen, Department of Orthodontics, Hanzeplein 1, 9700 RBGroningen, The Netherlands

**Keywords:** Metal organic framework, extracellular DNA, antibacterial, immunomodulation, wound healing

## Abstract

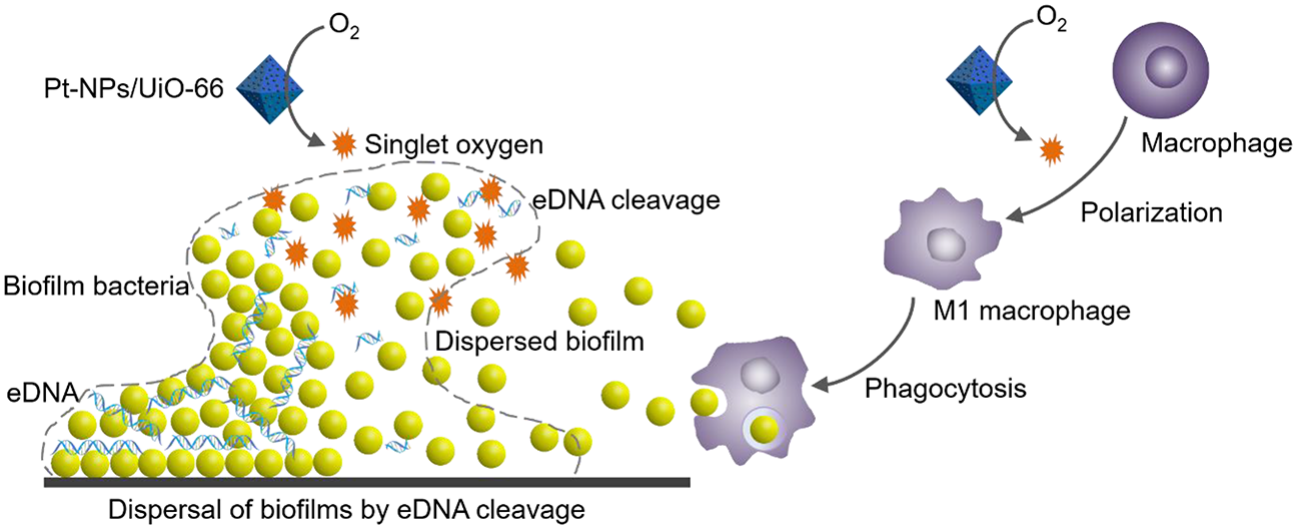

Eradication of infectious biofilms is becoming increasingly difficult due to the growing number of antibiotic-resistant strains. This necessitates development of nonantibiotic-based, antimicrobial approaches. To this end, we designed a heterocatalytic metal–organic framework composed of zirconium 1,4-dicarboxybenzene (UiO-66) with immobilized Pt nanoparticles (Pt-NP/UiO-66). Pt-NP/UiO-66 enhanced singlet-oxygen generation compared with Pt nanoparticles or UiO-66, particularly in an acidic environment. Singlet-oxygen generation degraded phosphodiester bonds present in eDNA gluing biofilms together and therewith dispersed biofilms. Remaining biofilms possessed a more open structure. Concurrently, Pt-NP/UiO-66 stimulated macrophages to adapt a more M1-like, “fighting” phenotype, moving faster toward their target bacteria and showing increased bacterial killing. As a combined effect of biofilm dispersal and macrophage polarization, a subcutaneous *Staphylococcus aureus* biofilm in mice was more readily eradicated by Pt-NP/UiO-66 than by Pt nanoparticles or UiO-66. Therewith, heterocatalytic Pt-NP/UiO-66 metal–organic frameworks constitute a nonantibiotic-based strategy to weaken protective matrices and disperse infectious biofilms, while strengthening macrophages in bacterial killing.

## Introduction

Prevention and treatment of infectious, bacterial biofilms using existing antibiotics is becoming increasingly difficult due to faster and faster development of antibiotic-resistant strains.^1,2^ Many nanoantimicrobials have been presented over recent years that have different mechanisms of action than current antibiotics.^3,4^ Antimicrobial, metal-based nanoparticles generally rely on metal-ion release^5,6^ or generation of reactive oxygen species (ROS)^7,8^ for enhanced bacterial killing. Smart, pH-responsive micelles^9,10^ and liposomes^11,12^ are used as antimicrobial nanocarriers to provide them with stealth properties facilitating self-targeting through the blood circulation toward an infectious biofilm. When composed of zwitterionic PCL-*b*-PQAE micelles,^13^ these micelles degrade important matrix components that glue an infectious biofilm together,^14^ such as polysaccharides, proteins, and eDNA. Degradation of these components leads to dispersal of infectious pathogens from a biofilm into the blood circulation.^15^ Once suspended in the blood circulation, it is crucial that pathogenic bacteria are rapidly killed, as they can cause sepsis or be carried through the blood circulation to cause infections elsewhere in the body.^16^ This constitutes a risk of approaches aimed to disperse infectious biofilms. This risk is enlarged by the speed at which bacteria gain resistance to antibiotics within due time after their market introduction.^1,2^ On the other hand, however, suspended bacteria are also more prone to clearance by host immune cells.^17^ Therewith, the clinical applicability of biofilm dispersants critically depends on an adequate immune cell response.

Macrophages constitute the first line of immune defense against invading pathogens and occur in a wide spectrum of different phenotypes,^18^ of which the M1-phenotype (also dubbed as the “fighting phenotype”) is specialized in eradicating invading, infectious pathogens. Its counterpart, at the opposite side of the polarization spectrum, is the anti-inflammatory M2-macrophage specialized in “fix-and-repairing” compromised tissue cells. In daily life, bleeding gums^19^ or open wounds^20^ are known to cause temporary, mild sepsis that is usually cleared by macrophages. However, dispersal of an infectious biofilm using dispersants yields a sudden, large increase in the number of pathogens in the blood, with which macrophages may not be sufficiently prepared to deal with. Therefore, infection-control strategies based on biofilm dispersal should be accompanied by appropriate measures to stimulate the natural immune response into an appropriate direction of macrophage polarization.

Here, we hypothesize that catalytic, noble metal nanoparticles immobilized in a metal–organic framework (MOF) have the ability to both disperse infectious biofilms and stimulate the immune defense. This hypothesis is based on the ability of Zn in MOFs with Zr as a transition metal^21^ to hydrolyze phosphodiester bonds through the generation of singlet oxygen.^22^ Phosphodiester linkages are abundant in DNA and usually extremely stable,^23^ while bacterially secreted eDNA is a pivotal glue in biofilm matrices for keeping its inhabitants together.^24,25^ Noble metal nanoparticles immobilized in MOFs may thus have the ability to disperse an infectious biofilm, making bacteria more susceptible for clearance by macrophages. In addition, generation of ROS has recently been demonstrated to elicit antitumor immunity through induction of pro-inflammatory M1-macrophage polarization.^26^ Thus, catalytic, noble metal nanoparticles immobilized in metal–organic frameworks may not only have the ability to disperse infectious bacterial biofilms but also, at the same time, stimulate the immune response toward an appropriate pro-inflammatory macrophage phenotype for optimal eradication of pathogens that have been dispersed from a biofilm.

The aim of this Article is to verify the above hypothesis. To this end, we first immobilized Pt nanoparticles (Pt-NP) in MOFs with Zr as a transition metal. A MOF with Zr as a transition metal (UiO-66) was chosen, because the Zr^⊕^ nodes can degrade organophosphate via Zr^4+^ cations, serving as a strong Lewis acid to activate and cleave the P–O bond in phosphodiesters through the generation of singlet oxygen.^22,27,28^ Pt nanoparticles were employed as noble metal nanoparticles based on a pilot experiment (Figure S1), indicating that the catalytic activity of Pt nanoparticles with more filled 5d orbitals was higher than that of other noble metals due to their higher transfer of electrons from UiO-66 to Pt nanoparticles. This causes the Zr^⊕^ node to become positively charged and increases the affinity between the MOF and organophosphates.^29^ Pt-NP/UiO-66 MOFs were characterized using Transmission Electron Microscopy (TEM), Energy-dispersive X-ray diffraction (XRD), and BET analysis. DNA cleavage, dispersal of *Staphylococcus aureus* Xen36 biofilms, macrophage polarization, and phagocytosis were studied *in vitro*. In addition, biofilm eradication, as induced by our heterocatalytic MOFs, was studied in a diabetic mouse model versus Pt nanoparticles and UiO-66 MOFs.

## Results

### Characterization of Pt-NP/UiO-66 MOFs

**Figure 1:**
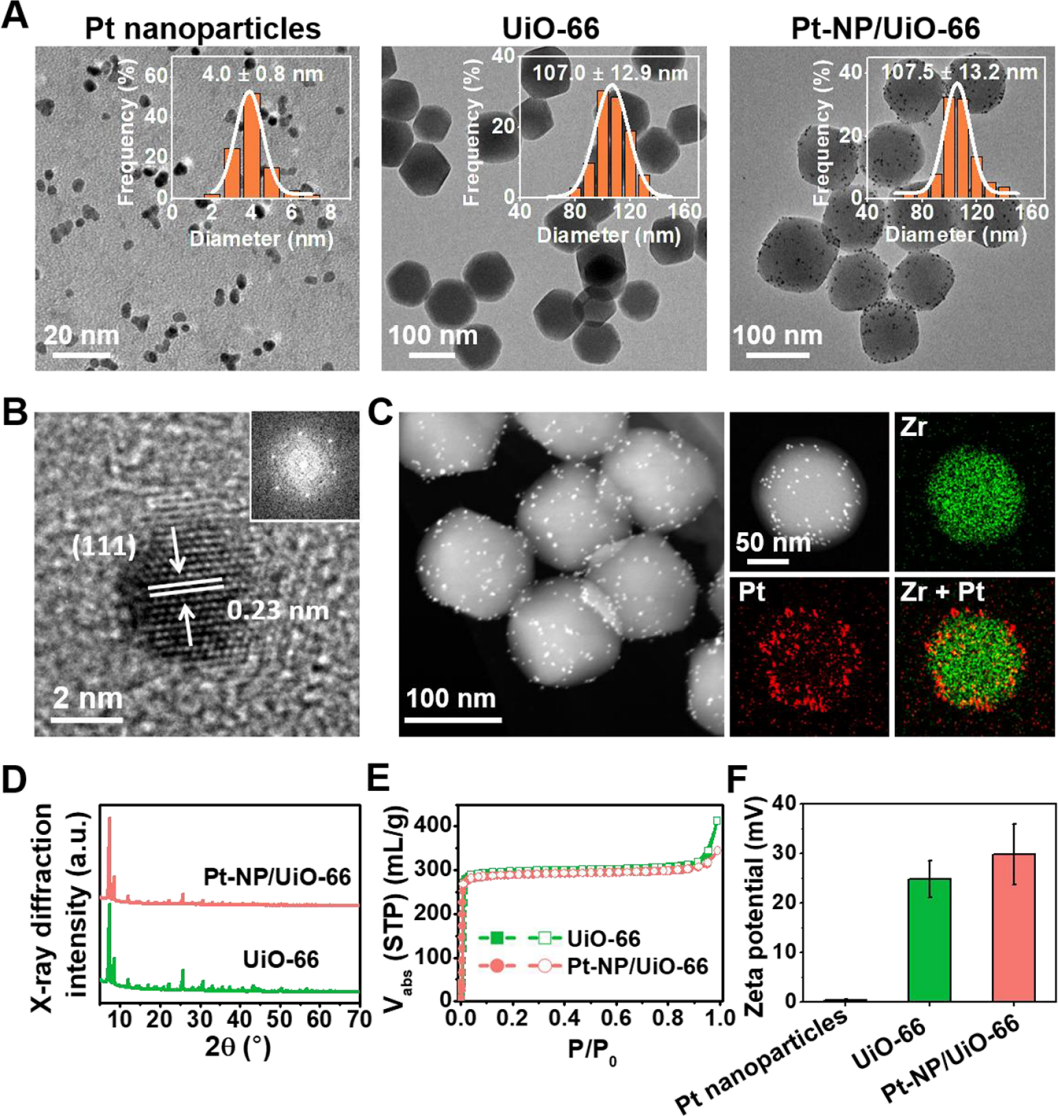
Characterization of Pt nanoparticles and MOFs. (A) TEM micrographs of Pt nanoparticles and UiO-66 and Pt-NP/UiO-66 MOFs. Insets represent the diameter distributions derived from the micrographs. Three TEM images were used to measure the diameters of the nanoparticles and MOFs, comprising a total of 200 nanoparticles and MOFs for each measurement. Diameters were plotted in histograms with 1 and 10 nm binning size for Pt nanoparticles and MOFs, respectively. Average diameters and standard deviations of the distributions were calculated by fitting a log-normal function to the data. (B) High-resolution TEM micrograph and corresponding fast Fourier transform (FFT) image (inset) of a Pt nanoparticle. (C) High-angle annular dark-field (HAADF), scanning TEM micrograph of single Pt-NP/UiO-66 MOFs, and corresponding energy-dispersive X-ray spectroscopy mapping of Zr and Pt. (D) X-ray diffraction patterns of UiO-66 and Pt-NP/UiO-66 MOFs. (E) Nitrogen (N_2_) sorption isotherms at 77 K of UiO-66 and Pt-NP/UiO-66 MOFs. The solid and open symbols represent adsorption and desorption, respectively. (F) Zeta potentials of Pt nanoparticles and UiO-66 and Pt-NP/UiO-66 MOFs in water. Data represent means over triplicate experiments with error bars indicating standard deviations.

The diameter of the Pt nanoparticles employed was 4 ± 1 nm, as derived from TEM micrographs (see Figure 1A). High-resolution TEM revealed that the nanoparticles were crystalline with a lattice spacing of 0.23 nm, while the corresponding FFT image indicated a hexagonal shape of the (111) plane (Figure 1B). UiO-66 MOFs had a diameter of 107 ± 13 nm (see also Figure 1A) with an octahedral shape. TEM demonstrated that Pt nanoparticles could be homogeneously immobilized on UiO-66 MOFs without aggregation (Figure 1A). Immobilization of Pt nanoparticles did not increase the diameter of Pt-NP/UiO-66 MOFs (108 ± 13 nm) as compared with UiO-66 MOFs. HAADF-STEM confirmed homogeneous distribution of Pt nanoparticles over the UiO-66 MOFs (Figure 1C). X-ray diffraction patterns of Pt-NP/UiO-66 and UiO-66 MOFs were similar (Figure 1D), indicating that the structure of UiO-66 was preserved upon immobilization of Pt nanoparticles. No identifiable peaks associated with Pt nanoparticles were observed, due to the low amount of Pt immobilized on the MOF (2 wt % as measured using inductively coupled plasma mass spectrometry). The relatively low number of Pt nanoparticles also did not impact the N_2_ sorption isotherms (Figure 1E) and UiO-66 and Pt-NP/UiO-66 had similar BET surface areas (897 and 873 m^2^/g, respectively). The zeta potential of the Pt nanoparticles (Figure 1F) was extremely small (+0.3 ± 0.2 mV), whereas both UiO-66 and Pt-NP/UiO-66 MOFs were highly positively charged (24.9 ± 3.8 and 29.8 ± 6.1 mV, respectively).

### Catalytic Activity of Pt-NP/UiO-66 MOFs and Generation of Singlet Oxygen

**Figure 2:**
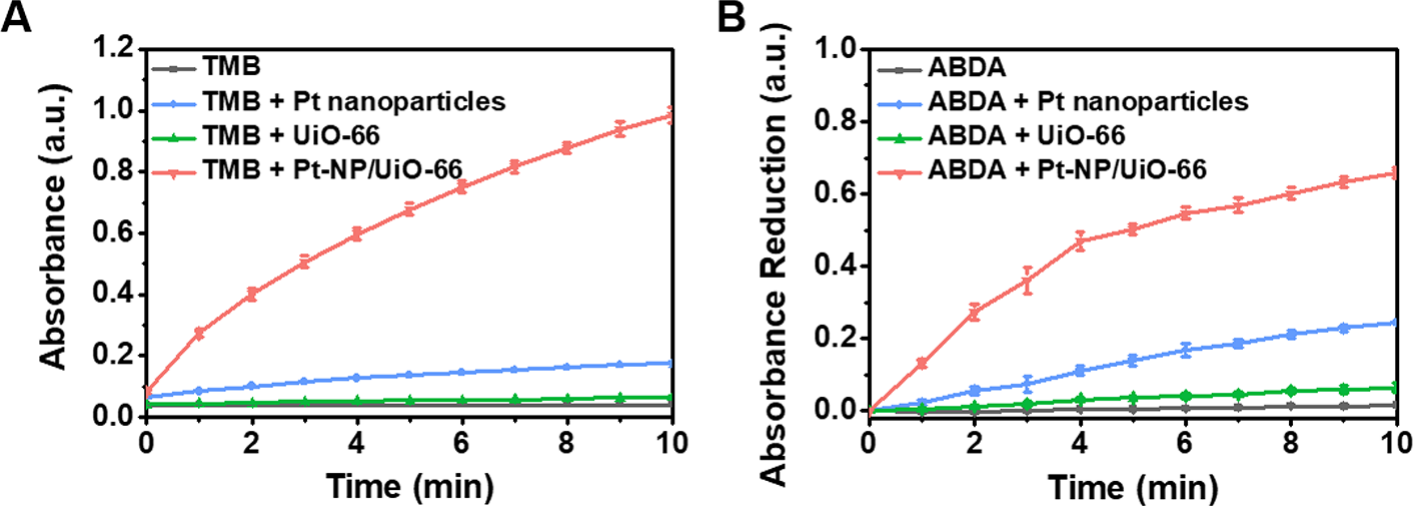
Catalytic activity and generation of singlet oxygen by Pt-NP/UiO-66 MOFs at acidic pH (pH 4). (A) The catalytic activity of suspended Pt nanoparticles and UiO-66 and Pt-NP/UiO-66 MOFs as a function of time, derived from TMB oxidation and expressed as the UV–vis absorbance at 652 nm (see Figure S2). The suspensions contained 0.4 μg/mL Pt nanoparticles or 20 μg/mL UiO-66 or Pt-NP/UiO-66 MOFs. (B) Generation of singlet oxygen measured from the oxidation of ABDA in the presence of Pt nanoparticles and UiO-66 and Pt-NP/UiO-66 MOFs as a function of time, derived from oxidation of ABDA into ABDA endoperoxide and expressed as a reduction in UV–vis absorbance at 400 nm (see Figure S5). Data represent means over triplicate experiments with error bars indicating standard deviations.

The catalytic activity of Pt-NP/UiO-66 MOFs was evaluated by studying the oxidation of 3,3′,5,5′-tetramethylbenzidine (TMB; see Figure S2A). Oxidation of TMB yields a blue color in acetate buffer (pH 4.0) (Figure S2B) with characteristic UV–vis absorption peaks at 370 and 652 nm (Figure S2C). Based on the UV–vis absorption spectra, Pt-NP/UiO-66 MOFs yielded faster and more extensive oxidation of TMB than Pt nanoparticles or UiO-66 MOFs in a low pH environment (Figure 2A), while in an environment with a physiological pH, the catalytic activity of heterocatalytic Pt-NP/UiO-66 MOFs based on oxidation of TMB is virtually absent (Figure S3). Thus, immobilization of Pt nanoparticles in the MOF is essential to establish electron transfer from the Zr^⊕^ node in the MOF to the Pt nanoparticle and therewith enhance catalytic activity, as a simple mixture of Pt nanoparticles and UiO-66 MOFs neither yielded faster nor more extensive oxidation of TMB (compare Figures 2A and S4A). Speculatively, Pt nanoparticles will be immobilized to the negatively charged carboxyl groups of the UiO-66 MOF through small electrostatic double-layer and ubiquitously present Lifshitz-Van der Waals attraction. Therewith, the Pt nanoparticle will be in the close vicinity of the Zr^⊕^ node in the MOF to facilitate electron transport from Zr^⊕^ node to Pt nanoparticles, required for the generation of singlet oxygen (see Figure S4B).

Generation of singlet oxygen by Pt-NP/UiO-66 MOFs was evaluated by studying the oxidation of 9,10-anthracenediylbis(methylene)dimalonic acid (ABDA) into ABDA endoperoxide (Figure S5A), generating a number of pronounced UV–vis absorbance peaks (Figure S5B). Based on the UV–vis absorption peak at 400 nm, Pt-NP/UiO-66 MOFs yielded much faster oxidation of ABDA than UiO-66 MOFs and Pt nanoparticles, indicating generation of higher amounts of singlet oxygen (Figure 2B).

### Degradation of Phosphodiester Bonds by Pt-NP/UiO-66 MOFs

**Figure 3:**
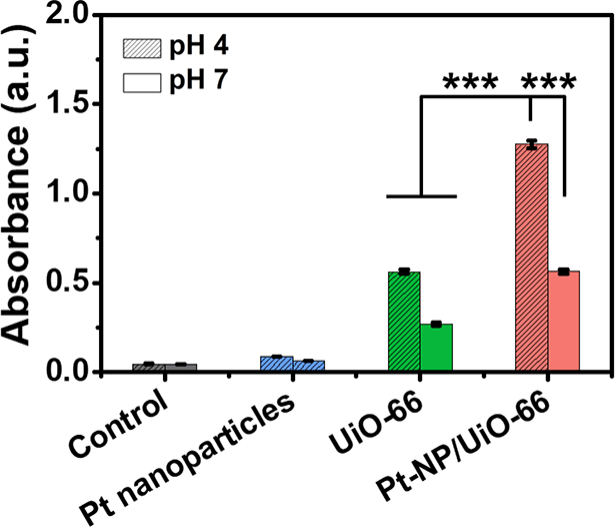
Degradation of phosphodiester bonds by Pt-NP/UiO-66 MOFs at pH 4 and 7. Degradation of the phosphodiester bond in BNPP by suspended Pt nanoparticles and UiO-66 and Pt-NP/UiO-66 MOFs was derived from the generation of nitrophenolate and the measurement of UV–vis absorbance at 400 nm (see Figure S6). The suspensions contained 2 μg/mL Pt nanoparticles or 100 μg/mL UiO-66 or Pt-NP/UiO-66 MOFs and were mixed for 5 min with BNPP (0.4 mM) in Tris buffer (50 mM). Shaded bars represent data pertaining to pH 4. Data represent means over triplicate experiments with error bars indicating standard deviations. *** indicates statistical significance (*p* < 0.001, two-tailed Student’s *t*-test) over the differences indicated by the spanning bar.

In order to evaluate the ability of Pt-NP/UiO-66 MOFs to degrade phosphodiester bonds in eDNA of biofilm matrices, bis(4-nitrophenyl)phosphate (BNPP) was used as a model molecule. DNA possesses similar phosphodiester bonds (Figure S6A) as BNPP (Figure S6B). The Zr^⊕^ node in UiO-66 catalyzes the hydrolysis of phosphodiester bonds to yield nitrophenolate (Figure S6C) with characteristic UV–vis absorbance peaks at 400 nm (Figure S6D). Pt nanoparticles had very low ability to degrade phosphodiester bonds but, when immobilized in Pt-NP/UiO-66 MOFs, yielded 2-fold higher degradation of phosphodiester bonds as UiO-66 MOFs without Pt nanoparticles at pH 4 as well as at pH 7 (Figure 3). However, degradation was significantly higher at pH 4 than at pH 7 (compare Figure S6D,E).

### Macrophage Polarization and Displacement as Stimulated by Pt-NP/UiO-66 MOFs

**Figure 4:**
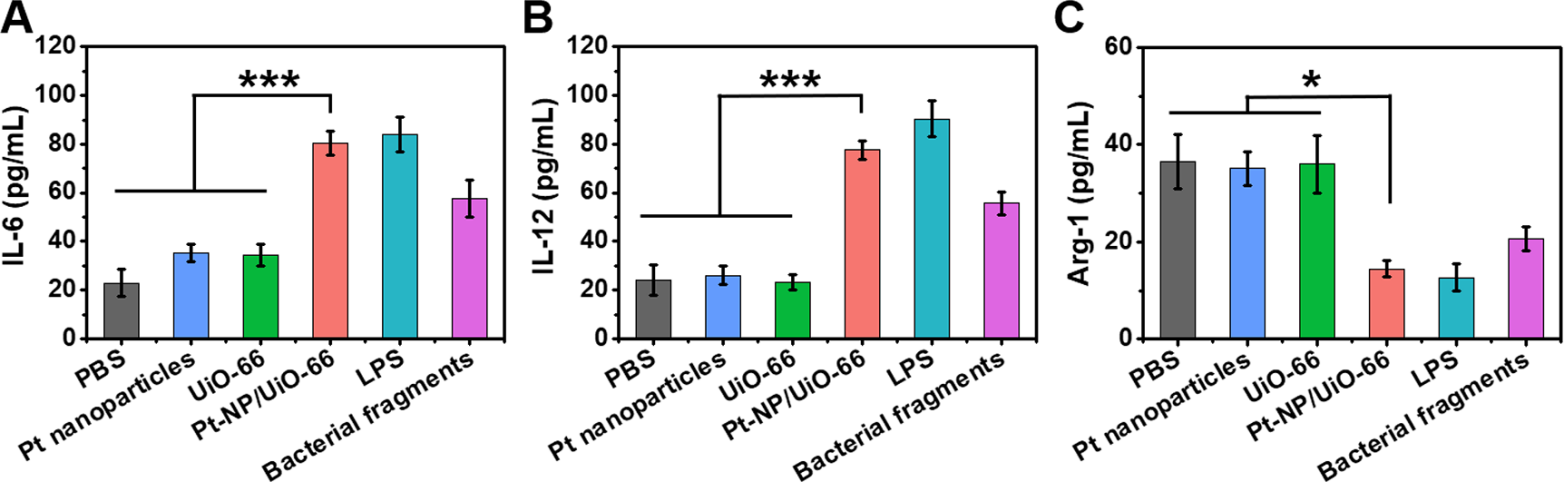
Cytokine secretion by macrophages upon exposure to Pt-NP/UiO-66 MOFs. Macrophages in DMEM-HG were exposed to Pt nanoparticles (8 μg/mL), UiO-66 (400 μg/mL), Pt-NP/UiO-66 MOFs (400 μg/mL), lipopolysaccharides (LPS, 10 μg/mL), or bacterial fragments (100 μg/mL) for 24 h. Secretion of IL-6, IL-12, and Arg-1 was measured using an enzyme-linked immunosorbent assay (see Figure S7 for calibration curves). (A) IL-6 secretion. (B) IL-12 secretion. (C) Arg-1 secretion. Data represent means over triplicate experiments with error bars indicating standard deviations. Error bars were taken from three parallel experiments. *p < 0.05 and ***p < 0.001 indicate statistical significance (two-tailed Student’s *t*-test) over the differences indicated by the spanning bars.

Next, the influence of Pt-NP/UiO-66 MOFs on the polarization of macrophages was determined by measuring macrophage secretion of IL-6 and IL-12, indicative of polarization toward the M1-phenotype and secretion of Arg-1, characteristic of the M2-phenotype. Exposure of macrophages to PBS, Pt nanoparticles, or UiO-66 MOFs yielded relatively low secretion of IL-6 (Figure 4A) and IL-12 (Figure 4B) but high secretion of Arg-1 (Figure 4C). This implied that macrophages tended toward the “fix-and-repair” M2-phenotype. Oppositely, exposure to Pt-NP/UiO-66 led to a relatively high secretion of IL-6 and IL-12 and low secretion of Arg-1, implying stimulation toward the “fighting” M1-phenotype. This pattern of macrophage polarization stimulated by Pt-NP/UiO-66, as displayed by three cytokines, was similar to that achieved by exposure to lipopolysaccharides or bacterial fragments (see also Figure 4). Although it can therefore be concluded that exposure to Pt-NP/UiO-66, lipopolysaccharides, or bacterial fragments induces (not necessarily the same) changes toward the M1-phenotype, it may not be concluded that these three differently stimulated macrophages are identical at the level of different M1 subphenotypes. Subphenotypic differences may exist that are not reflected by IL-6 and IL-12 and low secretion of Arg-1.

**Figure 5:**
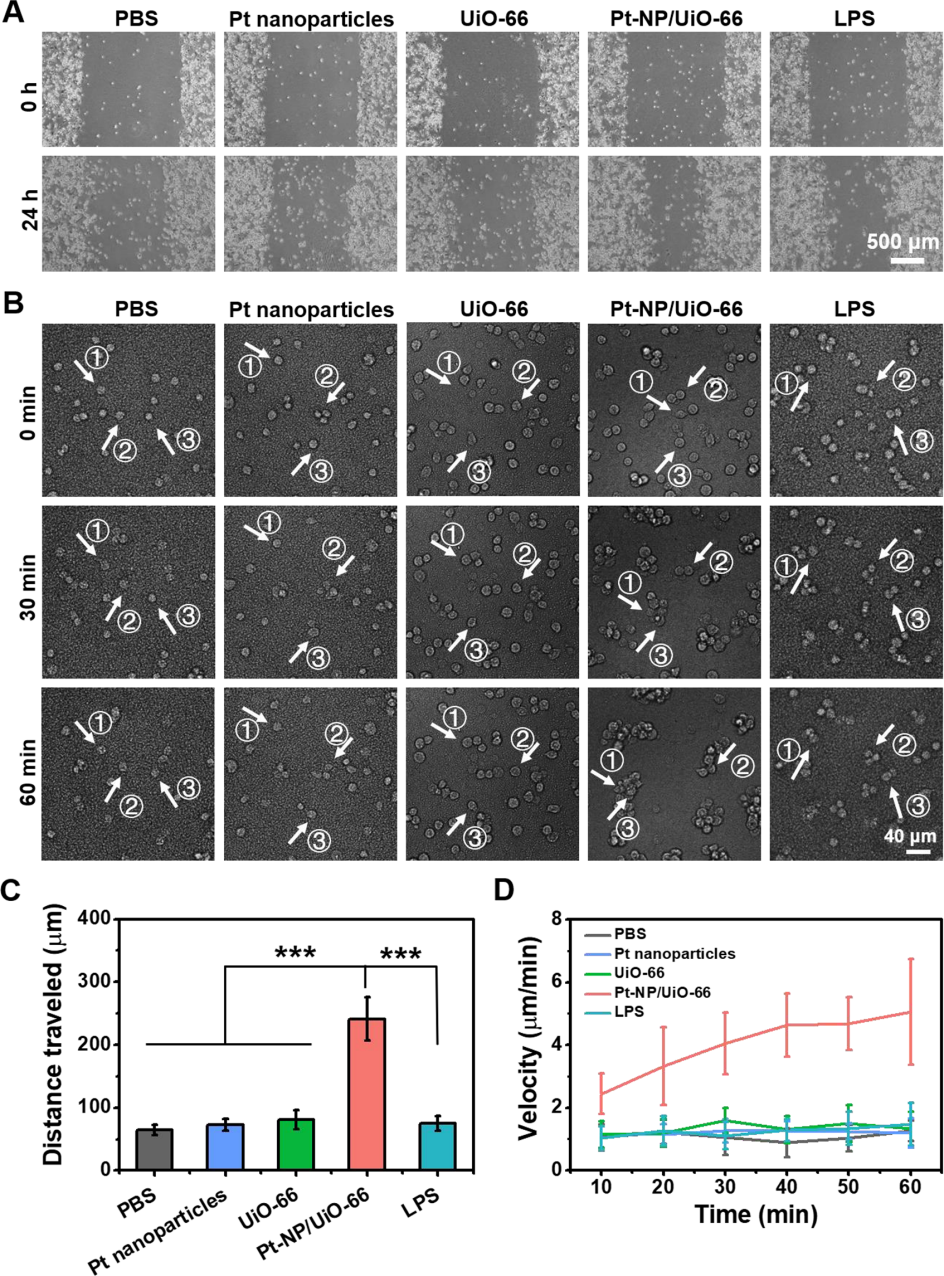
Displacement of macrophages on a nonbiological glass surface without adhering bacteria and on a biofilm surface in the presence of Pt-NP/UiO-66 MOFs. (A) 100,000 macrophages suspended in DMEM-HG supplemented with 8 μg/mL Pt nanoparticles or 400 μg/mL UiO-66 or Pt-NP/UiO-66 MOFs were sedimented on a circular glass coverslip in a confocal dish for 24 h after which a 1.5 mm scratch was made in the macrophage film. Bright field images were taken after another 24 h, showing that the scratch had closed for 49% and 53% by macrophages exposed to LPS or Pt-NP/UiO-66, respectively. (B) Biofilms grown for 24 h on a circular glass coverslip in a confocal dish were exposed for another 24 h to 8 μg/mL Pt nanoparticles or 400 μg/mL UiO-66 or Pt-NP/UiO-66 MOFs and suspended in TSB. Subsequently, after removal of TSB, 100,000 macrophages suspended in DMEM-HG were added. After sedimentation of the macrophages for 30 min, the migration distances were tracked by live imaging for 1 h. The numbers in the bright field images refer to individual macrophages (indicated by arrows) tracked over time. (C) Total distance traveled by macrophages on a surface with adhering bacteria. Data represent means over triplicate experiments with error bars indicating standard deviations. Error bars were taken from three parallel experiments. ***p < 0.001 indicates statistical significance (two-tailed Student’s *t*-test) over the differences indicated by the spanning bars. (D) Velocity of macrophage displacement on a surface with adhering bacteria in the presence of Pt-NP/UiO-66 MOFs as a function of time. Each data point represents the average over 20 macrophages in one experiment, with error bars indicating standard deviations.

Concurrent with stimulating macrophage polarization toward the fighting M1-phenotype, Pt-NP/UiO-66 MOFs and LPS increased the velocity at which macrophages moved over a nonbiological glass surface in absence of adhering bacteria (Figure 5A). In order to demonstrate possible differences in macrophage behavior in their fight against infecting bacteria, macrophage velocity was also studied on biological *S. aureus* biofilm surfaces after exposure to Pt nanoparticles, UiO-66 or Pt-NP/UiO-66 MOFs, or LPS (Figure 5B). Here, the distance traveled by adhering macrophages (Figure 5C) and their velocity (Figure 5D) were only larger in the presence of Pt-NP/UiO-66 MOFs but not in the presence of LPS. Note that macrophage velocity increased during the first 40 min of exposure to Pt-NP/UiO-66 MOFs and then leveled off, presumably because of the lower number of adhering bacteria left after phagocytosis. Likely, macrophage movement on a biofilm surface is driven by other mechanisms than on a nonbiological surface, such as the concentration gradients of chemo-attractants that are absent on a glass surface in the absence of adhering bacteria. The different behavior of macrophages exposed to Pt-NP/UiO-66 MOFs and LPS on a nonbiological surface in the absence of adhering bacteria versus a biofilm surface confirms our above suggestion that it may not be concluded on the basis of the secretion of three cytokines that macrophages are identical at the subphenotypic level.

### Effect of Pt-NP/UiO-66 MOFs on Biofilm Dispersal and Macrophage Action *in Vitro*

**Figure 6:**
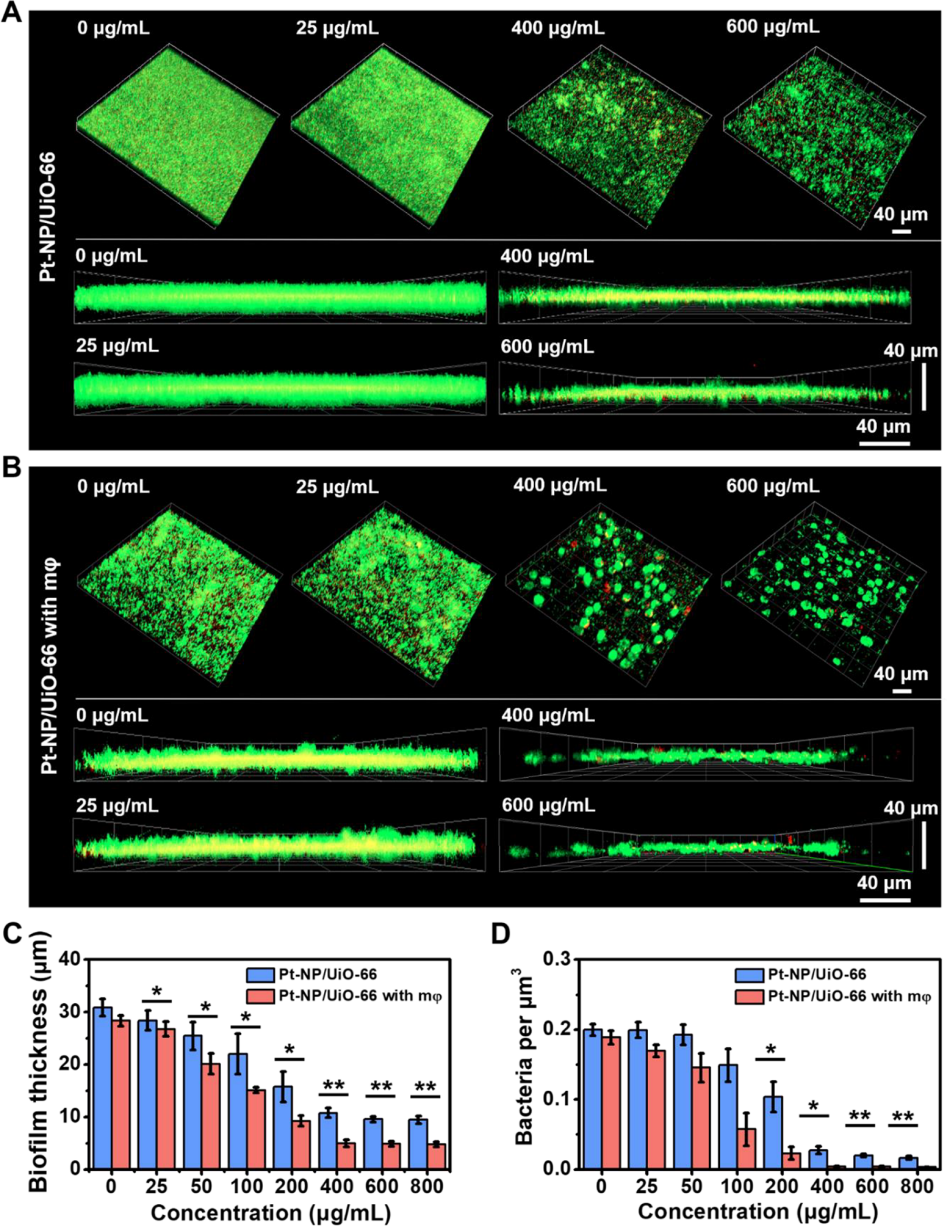
Dispersal of a 24 h *S. aureus* Xen36 biofilm upon 24 h of exposure to different concentrations of Pt-NP/UiO-66 MOFs in 2 mL of TSB and macrophage (mφ) action. Biofilms exposed to MOFs were stained with green-fluorescent SYTO9 and red-fluorescent propidium iodide for 3D confocal laser scanning microscopy (CLSM). In a separate experiment, MOFs pre-exposed staphylococcal biofilms were subsequently exposed to macrophages at MOF concentration up to 800 μg/mL. (A) 3D CLSM cross-sectional and overlayer images of staphylococcal biofilms exposed to selected concentrations of MOFs. (B) Similar as panel A, now after subsequent macrophage exposure. (C) Biofilm thickness, derived from 3D CLSM images as presented in panels (A) and (B), as a function of MOF concentration. (D) Similar as panel (C), now for volumetric bacterial densities. Volumetric bacterial densities in biofilms were calculated as the ratio of the number of CFUs cultured from a biofilm volume divided by the volume of biofilm derived from the 3D CLSM images in panels (A) and (B). Data represent means over triplicate experiments with separately prepared bacterial cultures and error bars indicating standard deviations. *p < 0.05 and **p < 0.01 indicate statistical significance (two-tailed Student’s *t*-test) over the differences indicated by the spanning bars.

In order to evaluate the net effect of Pt-NP/UiO-66 MOFs *in vitro*, 24 h old *S. aureus* Xen36 biofilms were exposed to Pt-NP/UiO-66 MOFs in the absence and presence of macrophages. Exposure of staphylococcal biofilms (see Figure 6A for CLSM images) to Pt-NP/UiO-66 MOFs in the absence of macrophages caused a concentration-dependent decrease in biofilm thickness (Figure 6C) as well as in the volumetric bacterial density in the biofilm (Figure 6D), indicative of a highly open biofilm structure after dispersal. Effects of biofilm exposure to Pt-NP/UiO-66 MOFs increased with MOF concentration up to 400 μg/mL. Higher concentrations of MOFs did not further stimulate biofilm dispersal. Decreases in biofilm thickness and bacterial volumetric densities after dispersal were far less when biofilms were exposed to Pt nanoparticles or UiO-66 MOFs (Figure S8), demonstrating the role of Pt nanoparticles immobilized in Pt-UiO-66 MOFs that act as a catalyst to speed up biofilm dispersal.

In order to evaluate whether biofilms with a more open structure, as left after dispersal, were more amenable to phagocytosis, biofilms pre-exposed to Pt-NP/UiO-66 MOFs were subsequently exposed to macrophages (see also Figure 6B). Based on the dispersal data, a Pt-NP/UiO-66 MOF concentration of 400 μg/mL was applied for pre-exposure. Macrophages were unable to decrease the thickness (see also Figure 6C) and volumetric bacterial density (see also Figure 6D) of staphylococcal biofilms in the absence of Pt-NP/UiO-66 MOFs pre-exposure, demonstrating the influence of a more open biofilm structure as achieved by Pt-NP/UiO-66 MOFs pre-exposure on the efficacy of phagocytosis.

### Biosafety of Pt-NP/UiO-66 MOFs

Biosafety of Pt-NP/UiO-66 MOFs was established *in vitro* and *in vivo*. Fibroblast and murine macrophage growth was not affected by the presence of Pt-NP/UiO-66 MOFs up to concentrations of at least 600 μg/mL (Figure S9), while hemolytic effects were also absent (Figure S10). In diabetic mice, neither blood biochemistry (Figure S11A) nor major organ tissues (Figure S11B) showed any signs of adverse effects of subcutaneous injection of Pt-NP/UiO-66 MOFs as compared with injection of PBS. Note the dose applied 600 μg/mL for establishing biosafety *in vivo* was 3 times higher than that applied for biofilm eradication (see below).

### Treatment of Infected Wound Using Pt-NP/UiO-66 MOFs in Mice

**Figure 7:**
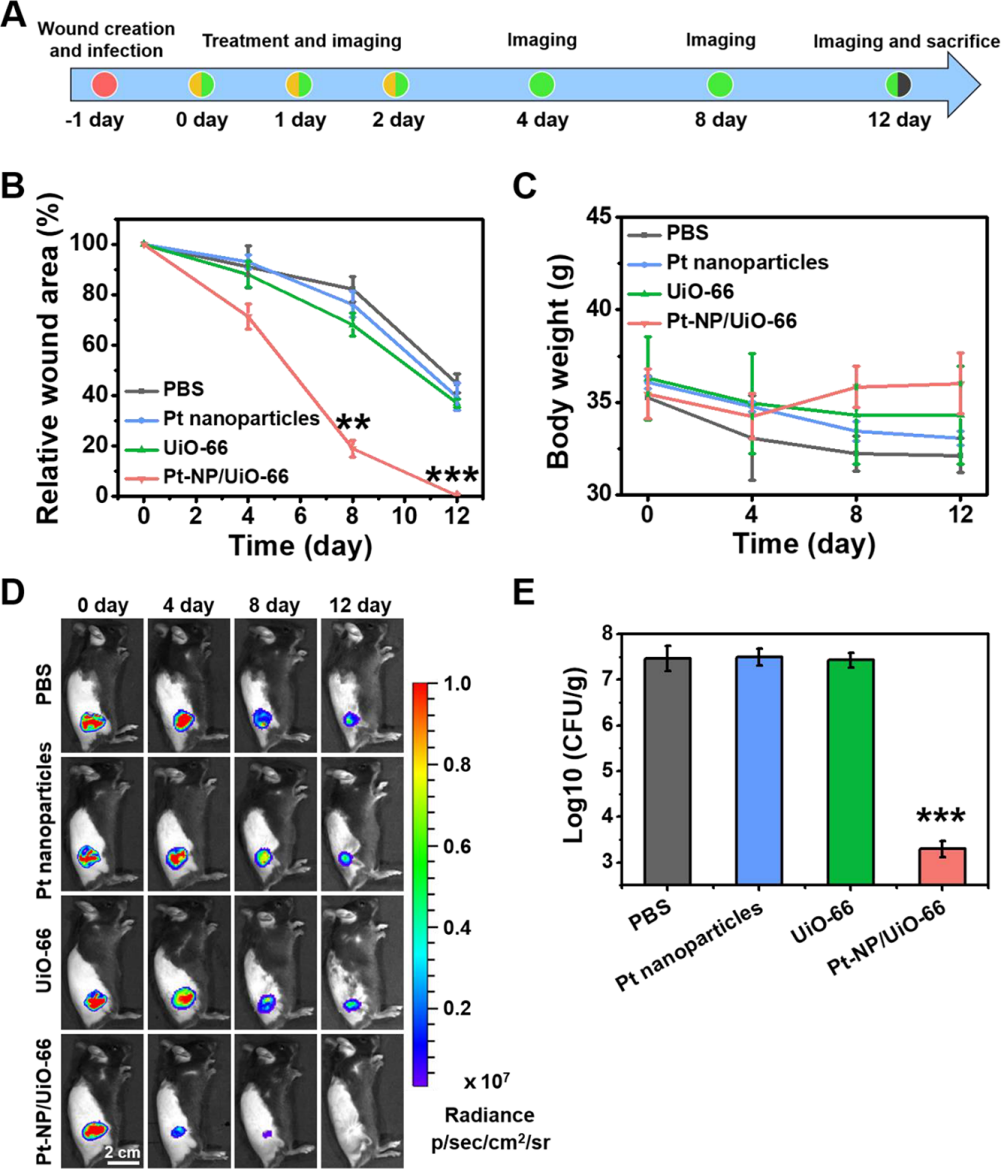
Healing of *S. aureus* Xen36 infected wounds in mice treated with Pt-NP/UiO-66 MOFs. (A) Time-line of *S. aureus* Xen36 infection, treatment, and imaging of the mice. Mice were treated by irrigation with 100 μL of PBS, 100 μL of 4 μg/mL Pt nanoparticles, and 100 μL of 200 μg/mL UiO-66 or Pt-NP/UiO-66 MOFs. (B) Infective wound area relative to the wound area on day 0. (C) Average body weight of mice after different treatments. (D) Bioluminescence images of infected wounds at different points in time after initiating infection. (E) The number of CFUs cultured from 100 mg of skin tissue taken from the initial wound site. Error bars were taken from three mice per group. *p*-values were calculated by the two-tailed Student’s *t*-test. **p < 0.01 and ***p < 0.001, compared with the UiO-66 group.

To study the net effect of Pt-NP/UiO-66 MOF use for *in vivo* biofilm eradication, a diabetic mouse model was applied. Skin wounds were infected with bioluminescent *S. aureus* Xen36 (dose 2 × 10^7^ per wound site) and treated by irrigation with 100 μL of PBS, Pt nanoparticles, or UiO-66 or Pt-NP/UiO-66 MOFs, starting 24 h after infection for three consecutive days (see scheme in Figure 7A). In the first instance, an evaluation was done by clinical assessment of symptoms of health and disease, i.e., visual inspection of the wound and monitoring of body weight and activity level of the mice. Visual inspection of the wound site confirmed full disappearance of the wound at day 12 upon treatment with Pt-NP/UiO-66 MOFs (Figure 7B). All groups of mice lost weight after bacterial infection, but the Pt-NP/UiO-66 MOFs treatment resulted in full recovery of body weight within the experimental period (Figure 7C). Recovery of body weight was concurrent with the return of a high activity level of the mice (Table S1), similar to that observed before entering the study. In groups not treated with Pt-NP/UiO-66 MOFs, the activity level of the mice was clearly less. On the microbiological side, bio-optical images of the infected wound site (Figure 7D) showed that bioluminescence persisted for at least 9 days after arresting treatment with PBS, Pt nanoparticles, or UiO-66 MOFs, i.e., day 12 of the experiment. Mice treated with Pt-NP/UiO-66 MOFs, however, hardly demonstrated bioluminescence arising from the wound site at day 8 of the experiment, and bioluminescence had fully disappeared at day 12 (see also Figure 7D). Also at sacrifice (experimental day 12), the number of staphylococcal CFUs cultured from homogenized tissue surrounding the wound area was 4 log units less than that cultured from tissues of mice treated with PBS, Pt nanoparticles, or UiO-66 MOFs (Figure 7E). Hematoxylin-eosin (H&E) staining of skin tissue of the wound area upon treatment with Pt-NP/UiO-66 MOFs showed a healthy epidermal morphology with a visible restoration of hair follicles within the experimental period (Figure S12), confirming the benefits of treatment using heterocatalytic Pt-NP/UiO-66 MOFs above the other treatment modalities investigated.

## Discussion

Here, we propose a dispersal strategy for the control of infectious biofilms, that is on the one hand based on degrading phosphodiester bonds with the eDNA of a biofilm matrix to disperse infecting pathogens and on the other hand based on strengthening the host immune response to kill bacteria dispersed in the blood circulation to prevent sepsis. This dual strategy was realized through the synthesis of heterocatalytic Pt-NP/UiO-66 MOFs generating singlet oxygen in the acidic environment of a biofilm^30^ or adhering bacteria^31^ and stimulating the polarization of macrophages toward a more M1-like phenotype migrating at higher velocity and killing bacteria with a high efficiency. Evidently, in this low pH environment, the short-lifetime of singlet oxygen (3–5 μs^32^), combined with the concentration at which it is generated, yields an intracellular ROS concentration that is within the limits of the intracellular antioxidant defense system that allows macrophages to balance their intracellular ROS concentration. Balanced ROS concentrations inside macrophages are crucial for their ability to kill internalized bacteria and stay alive themselves (see Figure S9B).^33^ Of clinical relevance, this dual strategy eliminates the danger of septic complications after dispersal of pathogens in the blood circulation that were contained in a biofilm before dispersal, relying solely on a strengthened immune system for bacterial killing without addition of antibiotics.

eDNA is an essential component of biofilm matrices and therewith a frequent target for dispersing biofilms.^34^ However, the phosphodiester bond in DNA is extremely resistant to hydrolysis and has a half-life of approximately 200 million years at neutral pH conditions (pH 7.0 and 25 °C).^22^ Although DNase as a naturally occurring enzyme and can hydrolyze DNA, DNase is highly sensitive to acids and alkali and easily deactivated at high temperature.^34^ Synthetic enzymes are generally much more stable.^35^ UiO-66 MOFs have excellent catalytic stability,^36^ and their catalytic activity is higher than that of most dinuclear molecular zinc complexes^37^ able to cleave phosphodiester linkages, such as Eu, Zn, Ho, and Yb.^22,37^ No structural changes were observed when immobilizing Pt nanoparticles in UiO-66 MOFs, while significantly enhancing the degradation of eDNA. This makes Pt-NP/UiO-66 MOFs an attractive alternative as a dispersant degrading eDNA above far less stable DNaseI.

Most studies on biofilm dispersal do not consider the hazards of planktonic pathogens in the blood circulation after dispersal.^38^ Bacteria in the blood circulation can lead to the formation of other biofilm infections elsewhere in the body.^39^ For example, implant-associated *Pseudomonas aeruginosa* biofilms dispersed by phosphodiesterase resulted in the accumulation of large amounts of bacteria in the spleen,^40^ induced bacterial colonization in the lungs causing pneumonia, and colonization of the middle ear, leading to acute otitis media.^16^ However, also once dispersed in the blood circulation itself, bacteria release endotoxins and induce a cytokine storm that can lead to a number of septic symptoms including death, as observed in mice after dispersal of a *P. aeruginosa* biofilm using glycoside hydrolase.^15^ Accordingly, the use of dispersants require simultaneous killing of dispersed biofilms to which end the immune system might arguably be considered most suitable in an era of growing antibiotic resistance among bacterial pathogens. Unfortunately, the immune system is not always prepared to deal with a sudden, high concentration of bacterial pathogens in the blood circulation as that after dispersal of an infectious biofilm. Pt-NP/UiO-66 MOFs stimulated polarization of macrophages toward the “fighting” M1-phenotype, similar to that achieved by exposure to lipopolysaccharides or bacterial fragments (Figure 4). This aided macrophages in their combat with dispersed pathogens and eliminated the need to use antibiotics to prevent septic symptoms in mice. Likely, the ability of Pt-NP/UiO-66 MOFs to generate singlet oxygen (Figure 2) promoted macrophage polarization. This suggestion is supported by observations that also singlet oxygen generated by ultrasmall, photosensitive Cu_2–*x*_Se nanoparticles under near-infrared irradiation^26^ stimulated macrophage polarization toward an M1-phenotype to enhance antitumor immunity. M1-polarization and biofilm eradication were also stimulated by erythrocyte-membrane encapsulated MoS_2_ nanodots^41^ and a hydrogel composed of poly(vinyl alcohol) modified with chitosan and polydopamine and using a red phosphorus nanofilm as an NO donor upon near-infrared irradiation.^42^ Both approaches neglected the consideration of potential septic symptoms, while the latter approach necessitated near-infrared irradiation, which provides a severe drawback compared to the use of our Pt-NP/UiO-66 MOFs, requiring no additional light triggering. Other approaches have advocated a preference for stimulating polarization toward the M2-phenotype to facilitate angiogenesis and blood vessel maturation in order to accelerate healing of the skin after chronic wound infection.^43^ Whereas this may be preferable in order to stimulate tissue healing, bacterial source elimination, particularly of pathogens involved in life-threatening sepsis, may be considered a first priority from a clinical perspective. Accordingly, the observation of a rapid increase in body weight and activity level in the group of mice treated with Pt-NP/UiO-66 and accompanying polarization of macrophage toward an M1 subphenotype may be more important than microbiological observations of reduced bioluminescence or reduced numbers of CFUs of which the clinical relevance is unknown.

## Study Limitations

Whereas this study successfully describes the use of a heterocatalytic Pt-NP-UiO66 MOF for the dispersal of infectious biofilms and stimulation of macrophages toward the M1-phenotype to yield more effective bacterial killing, we did not affirmatively demonstrate polarization to the M1-phenotype. For future research, this would require analysis of the possible secretion of more different cytokines. As a second study limitation, *in vivo* efficacy was only demonstrated for the healing of an infected wound. For wider applicability than infected wounds, it would be worthwhile to also apply the heterocatalytic MOFs developed toward the eradication of different types of infection, as caused by different bacterial strains and species than examined here. It may not be ruled out that other applications may require higher concentrations of MOFs to be administered, in which case it must be established that intramacrophage generation of ROS remains within the handling possibilities of the intracellular macrophage antioxidant defense system.

## Conclusion

A heterocatalytic Pt-NP/UiO-66 MOF was made that degraded eDNA in the matrix of acidic, infectious biofilms, causing its dispersal. Simultaneously, the MOF stimulated macrophages to adapt a more M1-like phenotype, facilitating their faster movement toward target bacteria and yielding more effective killing of bacterial pathogens. *In vivo* treatment of infected wounds with heterocatalytic Pt-NP/UiO-66 MOF, notably in absence of antibiotics, yielded fast healing without any septic complication, i.e., the common fear upon suddenly dispersing high numbers of bacterial pathogens in the blood circulation that were contained in a biofilm before dispersal. Therewith, this study shows that a dispersal strategy for the control of infectious biofilms based on a heterocatalytic Pt-NP/UiO-66 MOF can stimulate macrophages to combat and win the fight with infecting bacteria after dispersal without the aid of additional antibiotics.

## Materials and Methods

### Materials

Poly(vinylpyrrolidone) (PVP), ethylene glycol, sodium hexachlororhodate (Na_3_RhCl_6_), 1,4-dicarboxybenzene (H_2_BDC), *N*,*N*-dimethylformamide (DMF), and bis(4-nitrophenyl)phosphate (BNPP) were purchased from Sigma-Aldrich (St. Louis, MO, USA). Ethanol, glucose, and nitrophenolate were provided by Sinopharm Chemical Reagent Co., Ltd. (Shanghai, China). Ruthenium(III) chloride hydrate (RuCl_3_·3H_2_O), chloroplatinic acid (H_2_PtCl_6_), hexachloroiridium acid (H_2_IrCl_6_), acetone, palladium nitrate dihydrate Pd(NO_3_)_2_·2H_2_O, zirconium chloride (ZrCl_4_), acetic acid, 3,3′,5,5′-tetramethylbenzidine (TMB), and dimethyl sulfoxide (DMSO) were obtained from Shanghai Aladdin Biochemical Technology Co., Ltd. (Shanghai, China). 9,10-Anthracenediyl-bis (methylene) dimalonic acid (ABDA) was bought from Beyotime Institute of Biotechnology (Shanghai, China). Tryptone soy broth (TSB) was obtained from Hangzhou Microbial Reagent Co., Ltd. (Hangzhou, China). All chemicals were used without further purification. Ultrapure water (18.2 MΩ) was used throughout the experiments.

### Synthesis and Characterization of Pt-NP/UiO-66 MOFs

Pt nanoparticles^44^ and UiO-66 MOFs^45^ were prepared according to previously published methods. For the preparation of Pt-NP/UiO-66 MOFs, 6 mL of synthesized Pt nanoparticles (0.12 mg/mL) in water were added dropwise to 6 mL of UiO-66 MOFs (3 mg/mL) in absolute ethanol under vigorous stirring at room temperature. After stirring (500 rpm) at room temperature for 3 h, Pt-NP/UiO-66 MOFs were collected by centrifugation at 6000*g* for 10 min and washed twice with absolute ethanol and three times with water. Finally, Pt-NP/UiO-66 MOFs were resuspended in 6 mL of water for further use.

For transmission electron microscopy (TEM) characterization, Pt nanoparticles or UiO-66 or Pt-NP/UiO-66 MOFs suspended in absolute ethanol were drop-casted on carbon-coated copper grids. After evaporation of ethanol under ambient conditions, high-resolution TEM, high-angle annular dark-field scanning TEM (HAADF-STEM), and energy dispersive X-ray were performed using a TALOS 200× microscope operated at 200 kV (FEI, USA). Zeta potentials of the Pt nanoparticles and UiO-66 and Pt-NP/UiO-66 MOFs were measured in water using a Zetasizer Nano ZS (Malvern Instruments, UK). X-ray powder diffraction patterns were collected by a Philips X’pert PRO MPD diffractometer applying Cu Kα radiation (λ = 0.15406 nm). The operation voltage and current were kept at 40 kV and 40 mA.

### Catalytic Activity of Pt-NP/UiO-66 MOFs and Generation of Singlet Oxygen

The catalytic activity of Pt nanoparticles or UiO-66 or Pt-NP/UiO-66 MOFs was evaluated based on oxidation of 3,3′,5,5′-tetramethylbenzidine (TMB). To this end, 10 μL of TMB (50 mM) was mixed with 1 mL of acetate buffer (0.1 M acetic acid, 0.1 M sodium acetate, pH 4.0, 25 °C), and 10 μL of 40 μg/mL Pt nanoparticles or 2 mg/mL UiO-66 or Pt-NP/UiO-66 MOFs suspended in water were added and mixed. Immediately after mixing, 200 μL was taken and put into a clean 96-well plate and oxidation was monitored from UV–vis absorption spectra using a microplate reader (Synergy H1, BioTek, Winooski, VT, USA). Absorption spectra were recorded at 60 s intervals for a total duration of 600 s using the microplate reader, and the absorbance peak at 652 nm was used for quantitating catalytic activity on the oxidation of TMB.

The ability of Pt nanoparticles or UiO-66 or Pt-NP/UiO-66 MOFs to generate singlet oxygen was quantified based on oxidation of 9,10-anthracenediylbis(methylene)dimalonic acid (ABDA) into ABDA endoperoxide. To this end, 5 μL of ABDA (40 mM) in DMSO was added to 1 mL of 0.4 μg/mL Pt nanoparticles or 20 μg/mL UiO-66 MOFs or Pt-NP/UiO-66 MOFs in acetate buffer (pH 4) and immediately vortexed at room temperature. Then, 10 μL aliquots were taken from the reaction system at predetermined time intervals, and the UV–vis absorbance at 400 nm was measured using an UV–vis spectrometer (Nanodrop 2000c, Thermo Fisher Scientific) for quantitating singlet oxygen generation.

### Degradation of Phosphodiester Bonds by Pt-NP/UiO-66 MOFs

The ability of Pt nanoparticles or UiO-66 or Pt-NP/UiO-66 MOFs to degrade phosphodiester bonds was evaluated using bis(4-nitrophenyl)phosphate (BNPP), containing a high number of phosphodiester bonds, similar to that in DNA. To this end, 10 μL of BNPP (40 mM) in water and 50 μL of suspended Pt nanoparticles (40 μg/mL) or UiO-66 or Pt-NP/UiO-66 MOFs (2 mg/mL) were mixed in 1 mL of Tris-buffer (50 mM, pH 4.0 or 7.0) at room temperature. After 5 min, 200 μL of the mixed solution was transferred into a 96-well plate for measuring the UV–vis absorbance at 400 nm using a microplate reader for quantitating the ability to degrade phosphodiester bonds.

### Bacterial and Macrophage Culturing and Harvesting

Bioluminescent *S. aureus* Xen36 (PerkinElmer, Inc., Waltham, MA) was cultured on tryptone soy agar with 100 μg/mL kanamycin at 37 °C in ambient air. After 24 h, one colony was taken from the agar plate and inoculated in 8 mL of TSB with 100 μg/mL kanamycin at 37 °C for 24 h in ambient air. This preculture was diluted 1:20 in 8 mL of TSB and grown for 16 h at 37 °C. Staphylococci were collected by centrifugation at 4000*g* for 5 min and washed twice with PBS (Na_2_HPO_4_ 0.01 M, KH_2_PO_4_ 0.0018 M, NaCl 0.137 M, KCl 0.0027 M, pH 7.4). Finally, the bacteria were resuspended in 8 mL of PBS to a concentration of 3 × 10^9^ CFU/mL, as determined by plate counting in a series of separate experiments.

Murine macrophages J774A.1 (FH0329, Fu Heng biology, Shanghai, China) were cultured in Dulbecco’s modified Eagle Medium high glucose (DMEM-HG, Gibco, Thermo Fisher Scientific Inc., Waltham, U.S.A.) containing 10% fetal bovine serum (FBS, Gibco, Thermo Fisher Scientific Inc., Waltham, U.S.A.) and 1% penicillin/streptomycin (HyClone, Thermo Fisher Scientific Inc., Waltham, U.S.A.) at 37 °C in a humidified 5% CO_2_ incubator. At 70% confluency, macrophages were harvested by adding an EDTA-trypsin solution (Solarbio, Shanghai, China) and centrifuged at 500*g* for 10 min and resuspended in DMEM-HG.

### Biofilm Growth

Staphylococcal biofilms were grown on circular glass coverslips with a diameter of 18 mm in 12-well plates. To this end, 1 mL of *S. aureus* suspension in PBS (3 × 10^8^ CFU/mL) was added to the well and left for 1 h at 37 °C to allow bacterial sedimentation and adhesion. Next, the glass coverslip was washed with PBS and transferred into another 12-well plate containing 2 mL of TSB with 100 μg/mL kanamycin. The adhering bacteria were allowed to grow for 24 h at 37 °C to form a biofilm. Glass coverslips with biofilm were used for further experiments.

### Macrophage Polarization and Migration as Stimulated by Pt-NP/UiO-66 MOFs

To assess macrophage polarization, inflammatory cytokine expression was determined. To this end, macrophages suspended in DMEM-HG were seeded into a 6-well plate at a concentration of 1 × 10^5^ macrophages/well. After incubating for 24 h, the growth medium was replaced with fresh DMEM-HG containing 8 μg/mL Pt nanoparticles, 400 μg/mL UiO-66 or Pt-NP/UiO-66 MOFs, or 10 μg/mL lipopolysaccharides (LPS) and incubated for 24 h. After 24 h, growth medium was collected and centrifugated at 3000 rpm for 20 min to remove cell debris and nanoparticles. The expression levels of IL-6, IL-12, and Arg-1 in the supernatants were measured using an ELISA kit (Bio-Swamp, Wuhan, China) according to the manufacturer’s protocol. Absorbance values were measured at wavelength 450 nm using a microplate reader. For studying the effect of bacterial fragments on macrophage polarization, 5 mL of a bacterial suspension in PBS (1 × 10^7^ CFU/mL) was exposed to 400 μg/mL Pt-NP/UiO-66 MOFs for 8 h to kill and degrade the bacteria. After 8 h of exposure, the suspension was sonicated 5 times for 10 s (KQ-100 KDB, Kunshan Ultrasonic Instruments Co., Ltd., Kunshan, China) and centrifuged at 6000*g* for 5 min to remove Pt-NP/UiO-66 MOFs. The supernatant was collected and centrifuged at 60,000*g* for 1 h, and bacterial fragments were resuspended in 5 mL of PBS (see Figure S13). Suspensions with bacterial fragments were diluted 40 times with DMEM-HG before being incubated with macrophages (see above).

Migration of macrophages was studied by exposing a 24 h old staphylococcal biofilm (see above) during another 24 h to 8 μg/mL Pt nanoparticles, 400 μg/mL UiO-66 or Pt-NP/UiO-66 MOFs, or 10 μg/mL LPS. Subsequently, biofilms were gently washed once with PBS, and 2 mL of fresh DMEM-HG medium containing 100,000 macrophages was added. After 30 min, macrophage displacement was monitored over a time course of 1 h using a live-imaging, confocal laser scanning microscope (PerkinElmer UltraView VoX, Waltham, MA). The trajectories traveled by the macrophages were analyzed for the total displacement distance and velocity.

### Biofilm Eradication by Dispersal and Macrophage Action

*In vitro* biofilm eradication was studied by first exposing a 24 h old staphylococcal biofilm grown on a circular glass coverslip with a diameter of 18 mm in 12-well plates (for details see above) to fresh TSB with kanamycin containing different concentrations up to 800 μg/mL Pt-NP/UiO-66 MOFs. After 24 h, medium was discarded and biofilms were stained with SYTO9 and propidium iodide (LIVE/DEAD stain, Themo Fisher Scientific) for 30 min in the dark. Subsequently, cell wall damage was assessed using CLSM. In addition, staphylococci were retrieved from the surfaces by gently scraping the biofilm with a cell scraper, resuspended in 2 mL of PBS, and plated on tryptone soy agar after serial dilution to determine the numbers of viable staphylococci (CFUs) after 24 h of incubation.

In a second series of experiments, biofilms after exposure to Pt nanoparticles (8 μg/mL) and UiO-66 and Pt-NPs/UiO-66 MOFs (400 μg/mL) were grown for 24 h in the presence of macrophages (see also above), after which biofilms were analyzed using CLSM and plate counting (see above).

### *In Vitro* and *in Vivo* Biosafety

*In vitro* biosafety was assessed by culturing NIH 3T3 (ATCC CRL-1658) fibroblasts and murine macrophages J774A.1 in Petri dishes, filled with DMEM-HG containing 10% FBS and 1% penicillin/streptomycin at 37 °C in a humidified 5% CO_2_ incubator. At 70% confluency, the cells were harvested by adding an EDTA–trypsin solution, centrifuged at 500*g* for 10 min, and resuspended in DMEM-HG. The cells were seeded in 96-well plates (8000 cells, 100 μL per well) and grown for 24 h. The growth medium was removed, and fresh medium with different concentrations up to 600 μg/mL Pt-NP/UiO-66 MOFs was added. After another 24 h, cell viability was assessed using the CellTiter-Glo luminescent cell viability assay (Promega) according to the manufacturer’s instructions.

Hemolytic effects of Pt-NP/UiO-66 MOFs were evaluated using red blood cells (RBCs), drawn from diabetic mice (black mouse, C57BL/6J), supplemented with anticoagulant citrate dextrose. Blood (800 μL) was centrifuged at 500*g* for 5 min at 4 °C to collect RBCs after which RBCs were washed three times with PBS and suspended in PBS. Then, 0.1 mL of a RBC suspension was mixed with 0.9 mL of a Pt-NP/UiO-66 MOF suspension with different concentrations up to 600 μg/mL and incubated for 3 h at 37 °C. After centrifugation at 500*g* for 5 min, hemoglobulin absorbance in the supernatant was measured in a microplate reader at 540 nm. RBCs mixed with PBS or water were used as a negative and positive control, respectively. The relative hemolysis was calculated according to
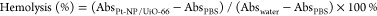
where Abs_Pt-NP/UiO-66_, Abs_PBS_, and Abs_water_ represents the absorbances of the respective hemoglobin in suspensions at 540 nm.

*In vivo* biosafety of Pt-NP/UiO-66 MOFs was evaluated in mice. Male C57BL/6J mice (8 weeks old) were provided by the Model Animal Research Center of Soochow University (Suzhou, China), and all experiments were performed in accordance with the guidelines and the approval of the Institutional Animal Care and User Committee at Soochow University (approval number 202112A0217). The back of the mice was shaved after which 100 μL of an 800 μg/mL Pt-NP/UiO-66 MOF suspension in PBS was injected subcutaneously into the right flank. As a control, 100 μL of PBS was injected. The injection was repeated three times with an interval of 24 h in between. At day 12 after the last injection, mice were sacrificed and blood was collected through the eye and left undisturbed for 30 min after which plasma was obtained by centrifugation at 500*g* for determination of routine blood parameters. Meanwhile, also major internal organs (heart, liver, spleen, lung, kidney) were collected for histological analysis. Organs collected were fixed in neutral buffered formalin, dehydrated with serial ethanol solutions, embedded in paraffin wax, sectioned into 4 μm slices, and stained with hematoxylin-eosin (H&E).

### Infected Wound Treatment by Pt-NP/UiO-66 MOFs in Mice

In order to make the mice more prone for infection, diabetes was inferred on the mice by fasting for 4 h, followed by intraperitoneal injection of streptozotocin (STZ) (120 mg/kg body weight in 10 mM citrate buffer, pH 4). During the subsequent 3 weeks, mice were fed with a high-fat diet (21.8 kJ/g, 60% of energy as fat) to stimulate diabetic type 2 mice.^46^

Wounds were created on the right flank of the mouse under anesthesia using intraperitoneally injected chloral hydrate (5%). Next, mice were shaven; the skin on the flank was lifted, and a curved scissor was used to create an open wound with a diameter of 15 mm. The wound was infected directly by dropping 100 μL (2 × 10^7^ CFUs) of *S. aureus* Xen36, and mice were immobilized for 30 min and individually housed for another 4 h. Mice with infected wounds were randomly divided into four groups of three mice. After 24 h (day 0), wounds were treated by dropping 100 μL of PBS as a control, 100 μL of Pt nanoparticles (4 μg/mL), 100 μL of UiO-66 MOFs (200 μg/mL), or 100 μL of Pt-NP/UiO-66 MOFs (200 μg/mL) on their wounds while immobilizing the mice during 30 min. Treatment was repeated at day 1 and day 2.

For bioluminescence imaging of the wounds, mice were anesthetized by being intraperitoneally injected with chloral hydrate (5%) and wound areas were imaged on days 0, 4, 8, and 12 using a bio-optical imaging system (Lumina III, Imaging System, PerkinElmer, 30 s exposure time, medium binning 1 F/stop, Open Emission Filter). Upon sacrifice at day 12, 100 mg of wound tissue was excised and homogenized in 1 mL of PBS for serial dilution, agar plating (see above), and CFU determination. For histology, wound skin sections were taken also upon sacrifice. Wound tissues in different groups were fixed in neutral buffered formalin, processed routinely into paraffin, sectioned into about 4 μm slices, and stained with hematoxylin-eosin (H&E). Tissue samples were examined under a digital microscope (IX73, OLYMPUS, Japan).

### Statistical Analysis

Data are expressed as means ± standard deviations (SDs). Statistical analysis was performed using SPSS v.16.0 software (SPSS Inc., USA). Statistical comparisons between multiple groups were conducted by a two-tailed Student’s *t*-test.

## Supporting Information Available

Catalytic activity of UiO-MOFs with different immobilized nanoparticles, SEM of bacterial fragments, catalytic activities of different particles based on oxidation of TMB, catalytic activities of Pt/UiO-66 MOFs based on oxidation of TMB as a function of pH, singlet oxygen generation based on oxidation of ABDA, degradation of phosphodiester bonds, calibration curves of cytokines, dispersal of biofilms by Pt-NP and UiO-66 MOFs, cytotoxicity of Pt-NP/UiO-66 MOFs, hemolytic effect of Pt-NP/UiO-66 MOFs, blood biochemistry and histological analyses of organ tissues of healthy mice after injection of Pt-NP/UiO-66 MOFs, histology of infected wound tissue after different treatments, and activity levels after different treatments of wound infection (PDF)
