# Supplementary material for: A Heterocatalytic Metal–Organic Framework to Stimulate Dispersal and Macrophage Combat with Infectious Biofilms
Source: ACS Nano. 2023 Jan 24;17(3):2328–40. doi: 10.1021/acsnano.2c09008 (PMC9933606; doi:10.1021/acsnano.2c09008)
Supplement: Supplementary file 1 — nn2c09008_si_001.pdf [file nn2c09008_si_001.pdf]

Supplemental Material

A heterocatalytic metal-organic-framework to stimulate dispersal and macrophage combat with infectious biofilms

Renfei Wu[1,2], Tianrong Yu[1,2], Sidi Liu[1,2], Rui Shi[1,2], Guimei Jiang[1,2], Yijin Ren[3], Henny C. van der Mei[2]*, Henk J. Busscher[2]*, Jian Liu[1]*

[1]Institute of Functional Nano and Soft Materials (FUNSOM), Jiangsu Key Laboratory for Carbon-Based Functional Materials and Devices, Joint International Research Laboratory of Carbon-Based Functional Materials and Devices, Soochow University, 199 Ren'ai Rd, Suzhou 215123, Jiangsu, P. R. China

[2]University of Groningen and University Medical Center Groningen, Department of Biomedical Engineering, Antonius Deusinglaan 1, 9713 AV Groningen, The Netherlands

[3]University of Groningen and University Medical Center of Groningen, Department of Orthodontics, Hanzeplein 1, 9700 RB, Groningen, The Netherlands

*Corresponding author.
Email: h.c.van.der.mei@umcg.nl (H.C.v.d.M.), h.j.busscher@umcg.nl (H.J.B.), jliu@suda.edu.cn (J.L.)

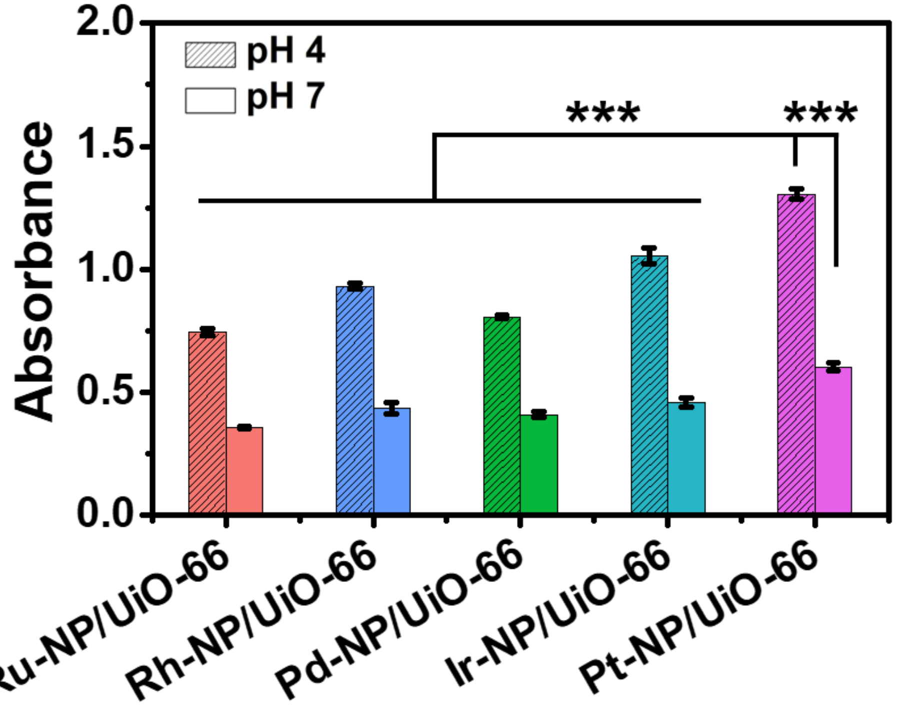

Figure S1. Catalytic activity of UiO-66 MOFs with immobilized nanoparticles of different noble metals at pH 4 and 7. Catalytic activity was measured by exposing bis(4-nitrophenyl)phosphate (BNPP, 0.4 mM) to different heterogeneous catalysts (100 μg/mL) for 5 min in Tris buffer (50 mM, pH 4 or 7). Catalytic activity was expressed as the UV-vis absorbance at 400 nm due to nitrophenolate, resulting from the oxidation of BNPP. Shaded bars represent data pertaining to pH 4. Data represent means ± standard deviations over three experiments. *** indicates statistical significance (p < 0.001, two-tailed Student's t-test) over the differences indicated by the spanning bars.

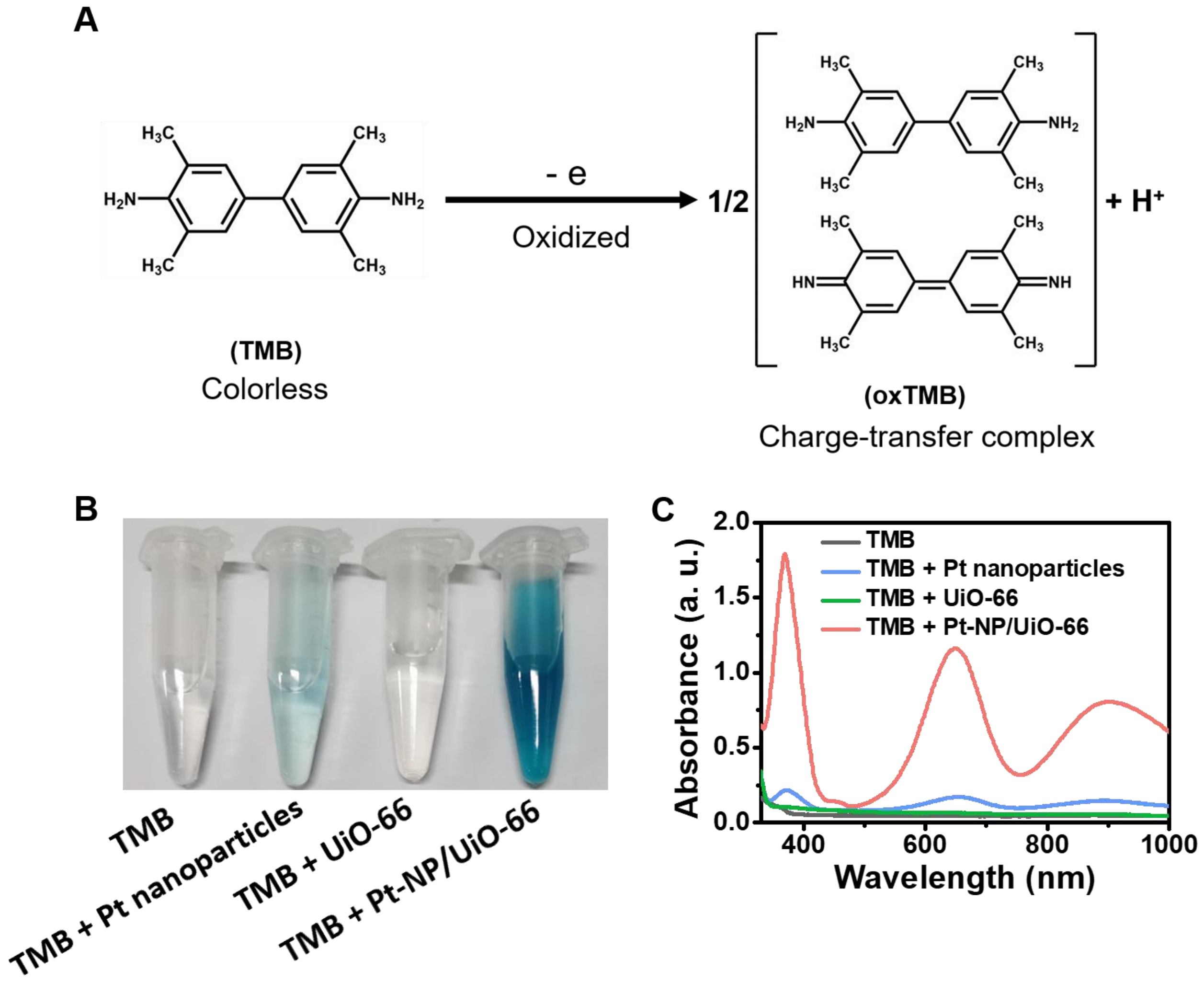

Figure S2. Measurement of catalytic activities based on oxidation of TMB at acidic pH (pH 4). (A) Oxidation of 3,3′,5,5′-tetramethylbenzidine (TMB). (**B**) Oxidation (600 s) of TMB (0.5 mM) in acetate buffer (1 mL, 0.1 M pH 4) at room temperature yields a distinct blue color in presence of 20 µg/mL Pt/UiO-66 MOFs, but this catalytic activity does not occur in presence of 0.4 µg/mL Pt nanoparticles or 20 µg/mL UiO-66 MOFs. (**C**) UV-vis absorption spectra of the suspensions shown in panel (B).

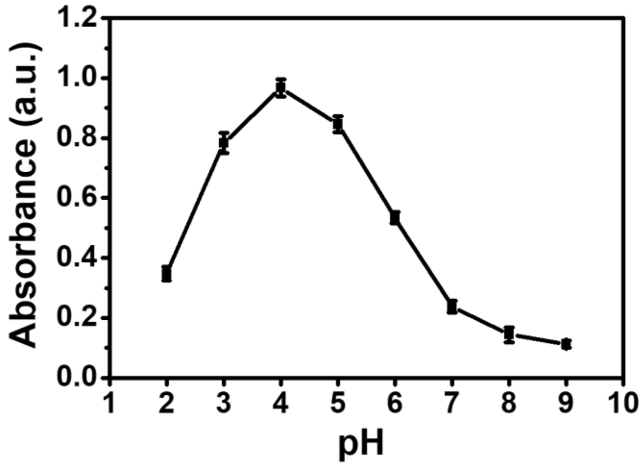

Figure S3. Catalytic activities of Pt-NP/UiO-66 MOFs based on oxidation of TMB as a function of pH. (for further details see **Figure S2**). Data represent means over triplicate experiments with error bars indicating standard deviations.

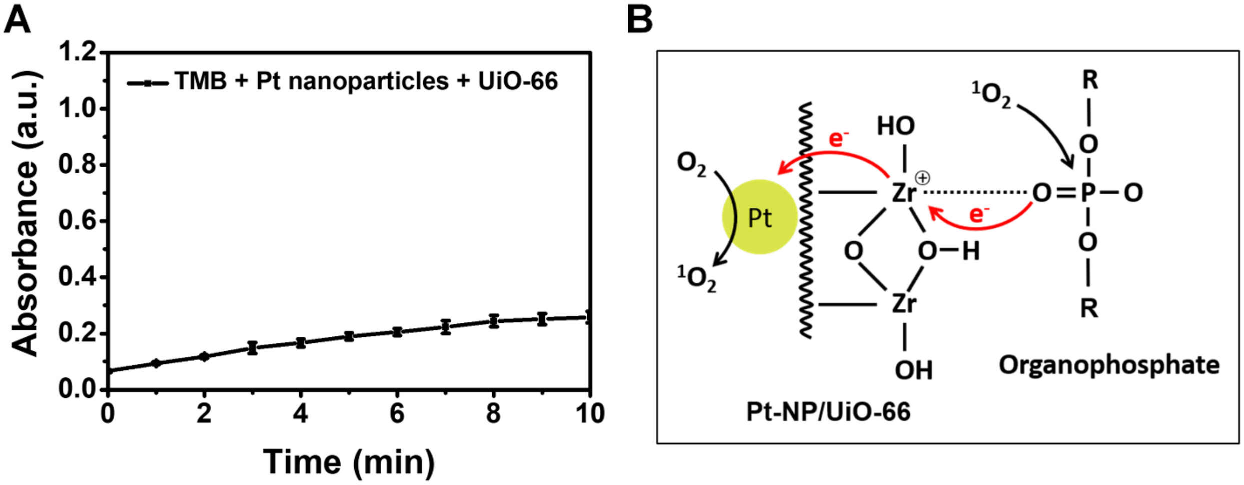

Figure S4. Essence of immobilization of Pt nanoparticle in the UiO-66 MOF. (A) Catalytic activity of a mixture of suspended Pt nanoparticles and UiO-66 MOFs at acidic pH (pH 4), derived from TMB oxidation and expressed as the UV-vis absorbance at 652 nm (see **Figure S2**). The suspensions contained 0.4 µg/mL Pt nanoparticles and 20 µg/mL UiO-66 MOFs. Data represent means over triplicate experiments with error bars indicating standard deviations. **(B)** Transfer of electrons from the Zr⊕ node in a UiO-66 MOF to fill the 5d orbitals of the Pt noble metal nanoparticles yields catalytic activity, speeding up singlet oxygen generation.

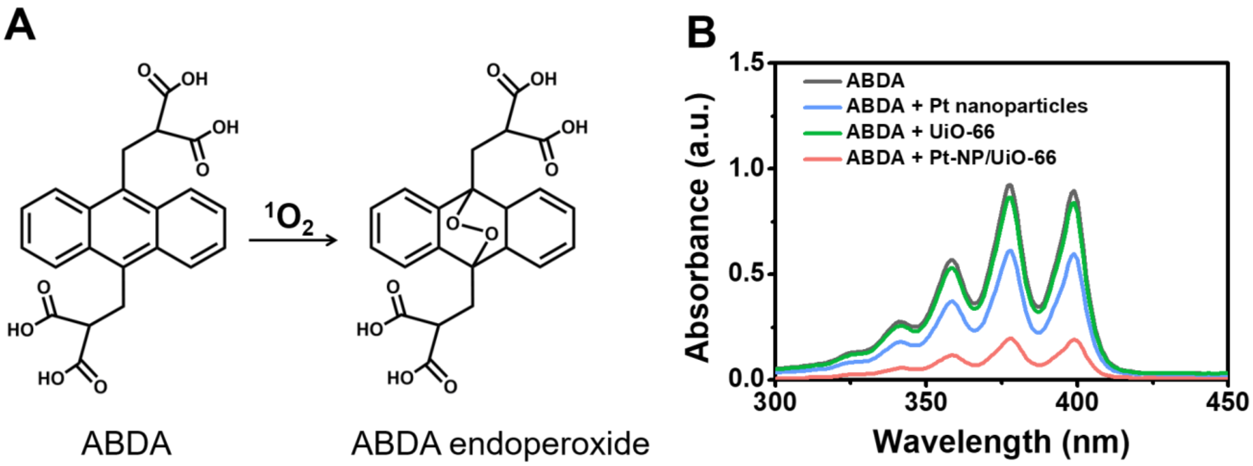

Figure S5. Measurement of singlet oxygen generation based on oxidation of 9,10-anthracenediyl-bis(methylene)dimalonic acid (ABDA) at acidic pH (pH 4). (**A**) Oxidation of ABDA by singlet oxygen into ABDA endoperoxide. (**B**) UV-vis absorption spectra of suspensions containing 0.4 µg/mL Pt nanoparticles or 20 µg/mL UiO-66 or Pt-NP/UiO-66 MOFs after 10 min mixing with an ABDA solution (1 mL, 0.2 mM).

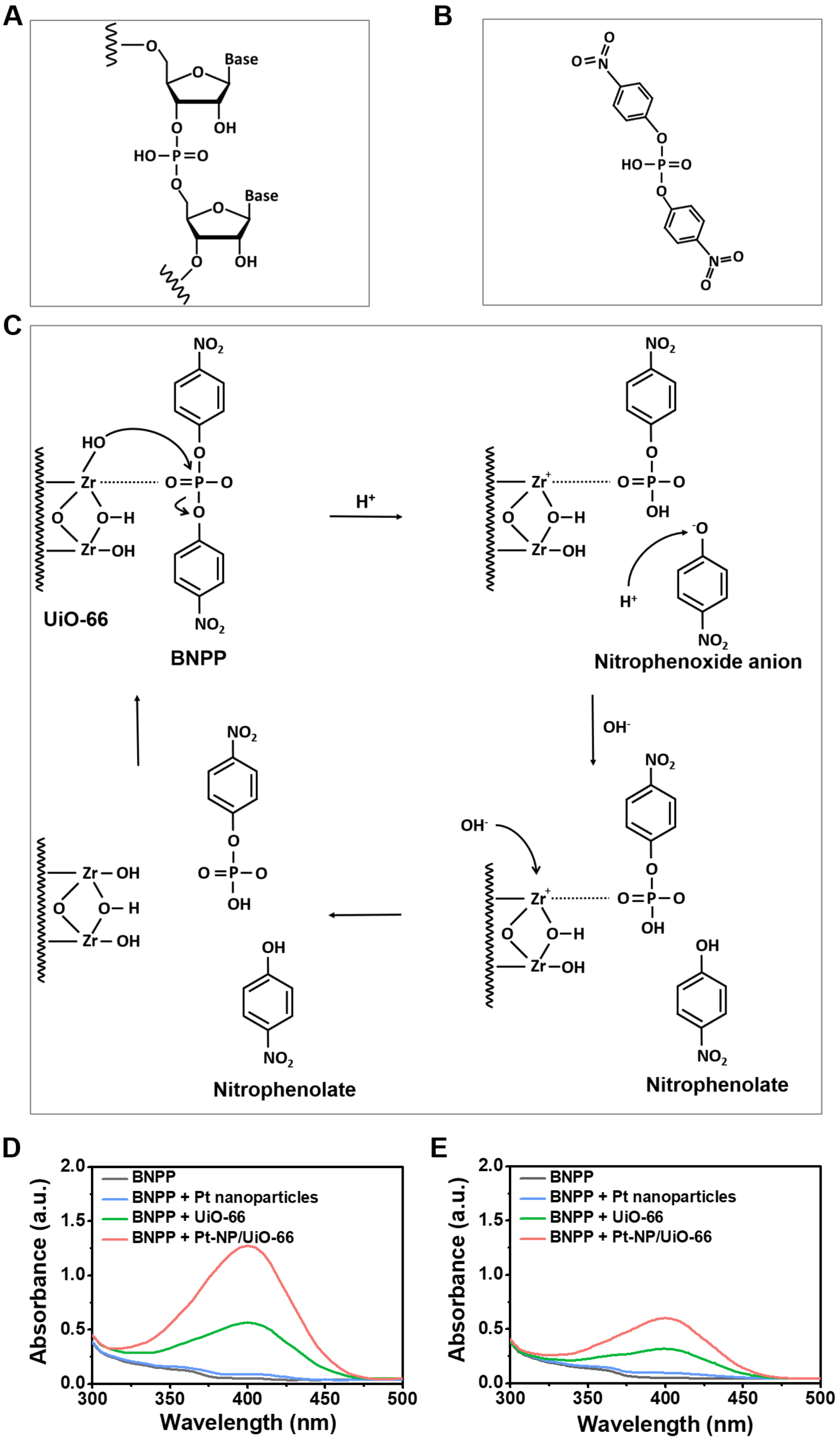
A
B
C
D
E
Base
OH
HO–P=O
NO_2
UiO-66
BNPP
Nitrophenoxide anion
Nitrophenolate
H^+
OH^-
Absorbance (a.u.)
Wavelength (nm)
BNPP
BNPP + Pt nanoparticles
BNPP + UiO-66
BNPP + Pt-NP/UiO-66

Figure S6. Measurement of the degradation of phosphodiester bonds in bis(4-nitrophenyl)phosphate (BNPP). (A) Phosphodiester bond in a DNA backbone. **(B)** Phosphodiester bond in BNPP. **(C)** Degradation pathway of BNPP by the Zr6-based node in UiO-66 MOF. The Zr^{+} node strongly attracts electrons from phosphate groups in BNPP, which lead to the activation of the phosphodiester linkage for the nucleophilic attack by a hydroxide ion. Finally, in the breakdown of the intermediate, the -OH of nitrophenolate is removed from the phosphorus atom through the scission of the P-O bond.[1] In addition, electrons can be passed on from Zr⊕ node to an immobilized Pt nanoparticle increasing the positive charge of the node and enhancing catalytic activity (see **Figure S4B**). **(D)** UV-vis absorption spectra of nitrophenolate, obtained after mixing suspensions containing 2 μg/mL Pt nanoparticles or 100 μg/mL UiO-66 or Pt-NP/UiO-66 MOFs after 5 min mixing with BNPP (0.4 mM) in Tris buffer (50 mM, pH 4). **(E)** Same as panel (D) but now for pH 7.

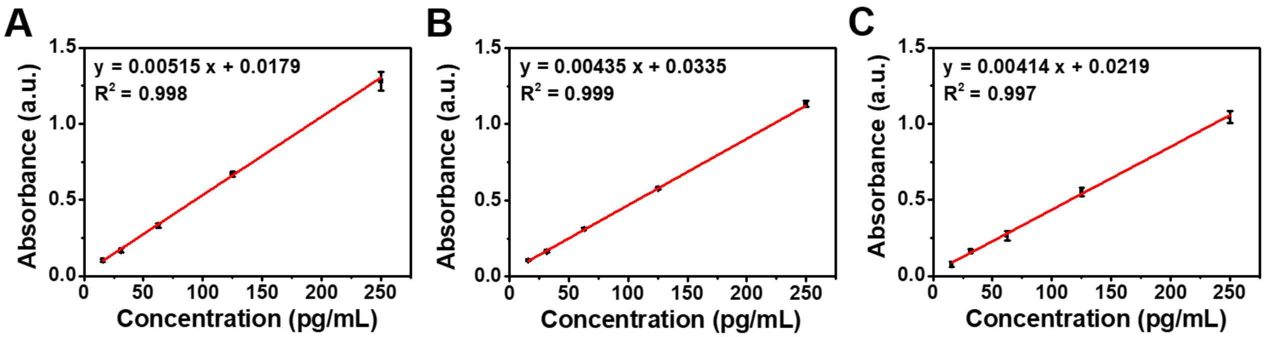

Figure S7. Calibration curves of the absorbance of cytokine solutions as a function of cytokine concentration in an enzyme-linked immunosorbent assay. Absorbances were measured at a wavelength of 450 nm using a microplate reader. (**A**) Absorbance as a function of IL-6 concentration. (**B**) Absorbance as a function of IL-12 concentration. (**C**) Absorbance as a function of Arg-1 concentration. Data represent means over triplicate experiments with error bars indicating standard deviations.

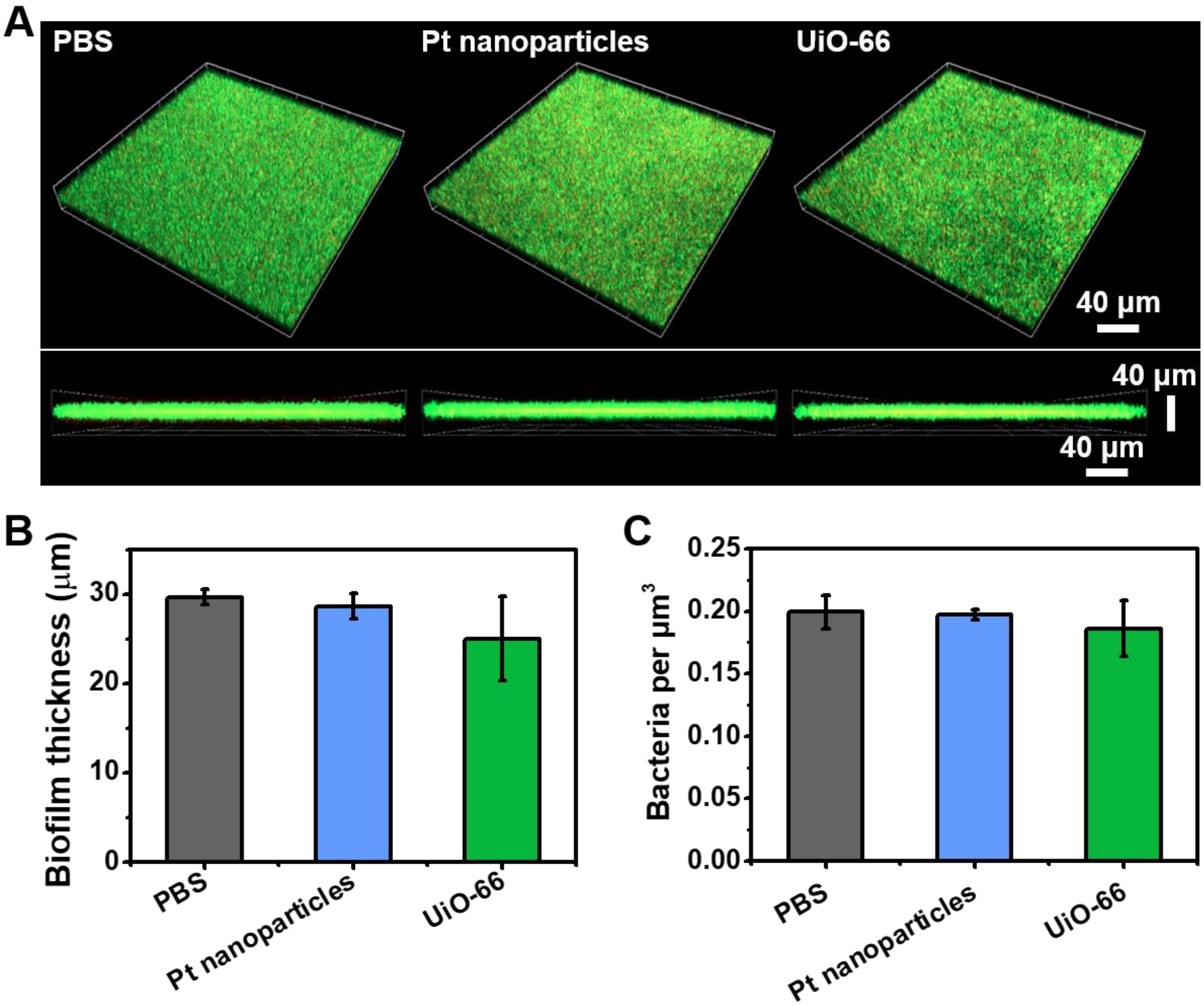

Figure S8. Dispersal of a 24 h *S. aureus* Xen36 biofilm upon 24 h exposure to Pt nanoparticles (8 μg/mL) or UiO-66 MOFs (400 μg/mL) in 2 mL TSB. MOFs exposed biofilms were stained with green-fluorescent SYTO9 and red-fluorescent propidium iodide for 3D confocal laser scanning microscopy (CLSM). (**A**) 3D CLSM cross-sectional and overlayer images of Pt nanoparticles or UiO-66 MOFs exposed biofilms. **(B)** Biofilm thickness, derived from 3D CLSM images presented in panel (A). **(C)** Volumetric bacterial densities in biofilms were calculated as the ratio of the number of CFUs cultured from a biofilm volume, divided by the volume of biofilm derived from the 3D CLSM images in panel (A). Data represent means over triplicate experiments with separately prepared bacterial cultures and error bars indicating standard deviations.

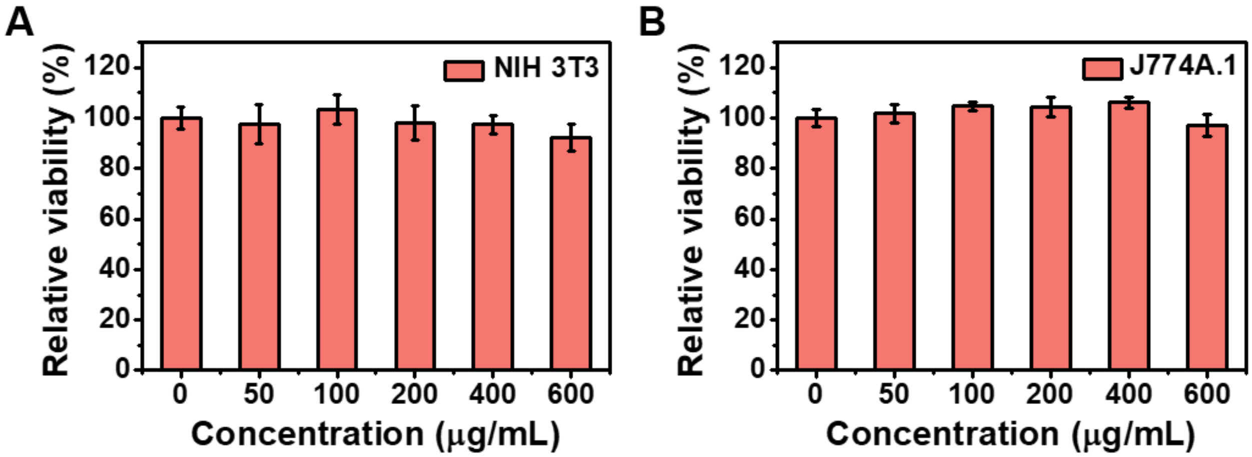

Figure S9. Influence of Pt-NP/UiO-66 MOF on tissue cell viability. (A) Relative viability of NIH 3T3 fibroblasts after 24 h growth in the presence of different concentration of Pt-NP/UiO-66 MOFs. **(B)** Same as panel A, now for macrophages. Data represent means over five experiments with separately prepared cellular cultures and error bars indicating standard deviations.

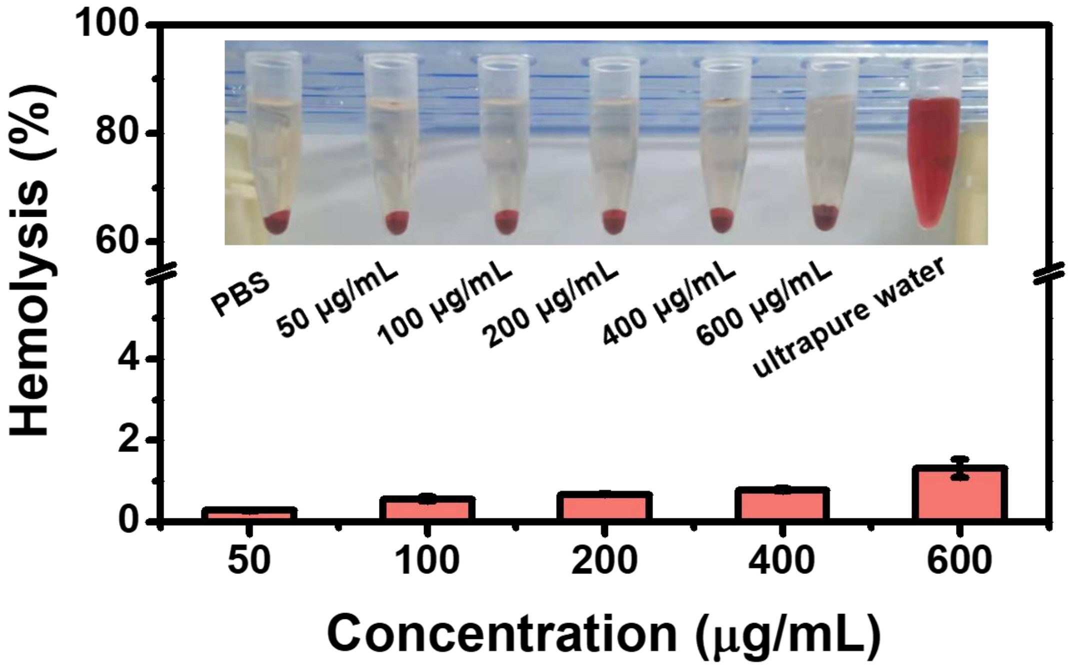

Figure S10. Hemolytic effects of Pt-NP/UiO-66 MOFs. Relative hemolysis of mouse red blood cells after 3 h exposure to different concentrations of Pt-NP/UiO-66 MOFs at 37ºC. Relative hemolysis was derived from UV absorbance at 540 nm, setting hemoglobin absorption of cells exposed to ultrapure water as 100%. Data represent means over triplicate experiments with error bars indicating standard deviations.

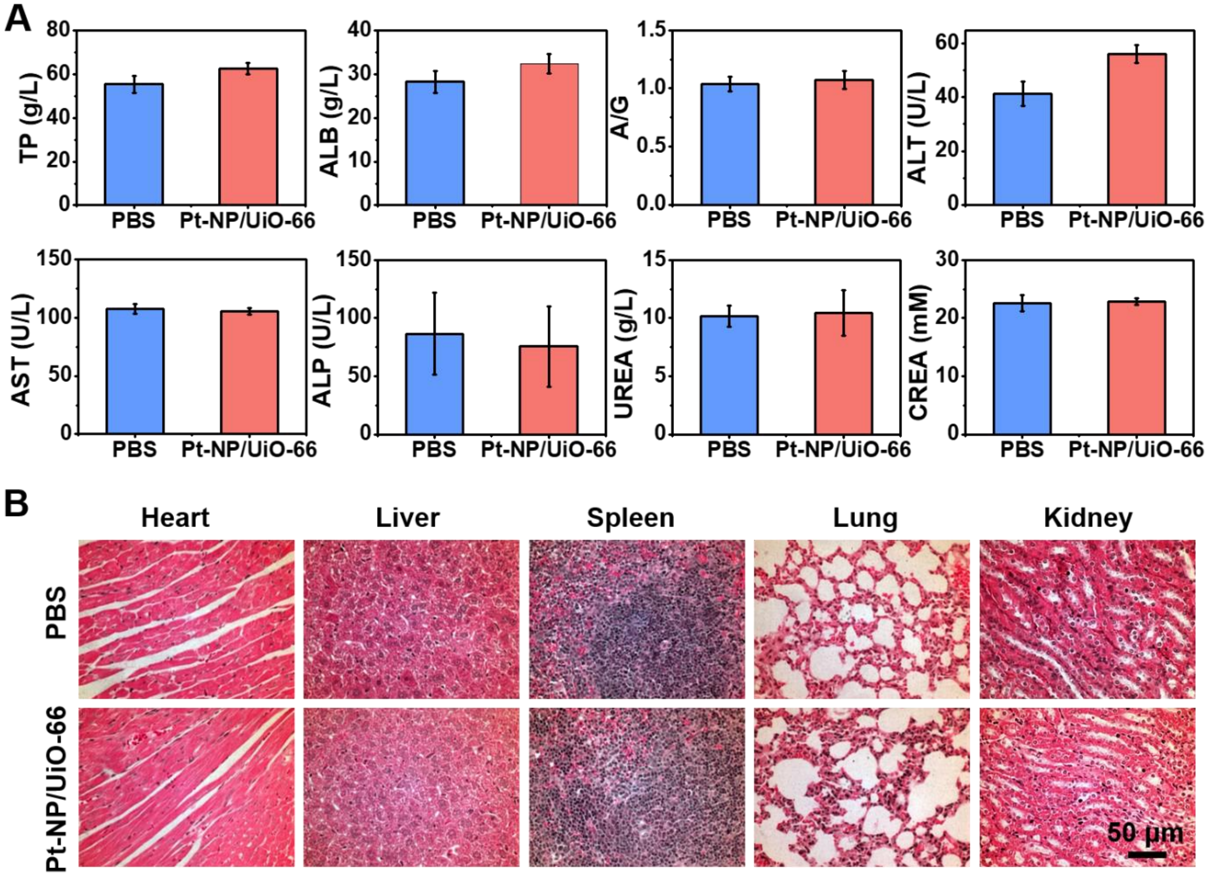

Figure S11. Blood biochemistry and histological analyses of major organ tissue of healthy, i.e. non-infected, mice after injection of Pt-NP/UiO-66 MOFs. Mice were subcutaneously injected with 100 µL of a Pt-NP/UiO-66 MOF suspension (800 µg/mL) in PBS or 100 µL PBS. The injection was repeated three times with an interval of 24 h. At day12 after the first injection, blood was extracted through the eye and used for biochemical assays. After sacrifice at day 12, internal organs were collected for histological analysis and stained by hematoxylin and eosin (H&E). (**A**) Blood parameters: total protein (TP), albumin (ALB), the ratio of albumin and globulin (A/G), alanine transaminase (ALT), aspartate transaminase (AST), alkaline phosphatase (ALP), urea nitrogen (UREA), and creatinine (CREA). Error bars indicate standard deviations over three mice in each group. (**B**) Histology of internal organs (heart, liver, spleen, lung, kidney) from the mice treated with Pt-NP/UiO-66 MOFs or PBS.

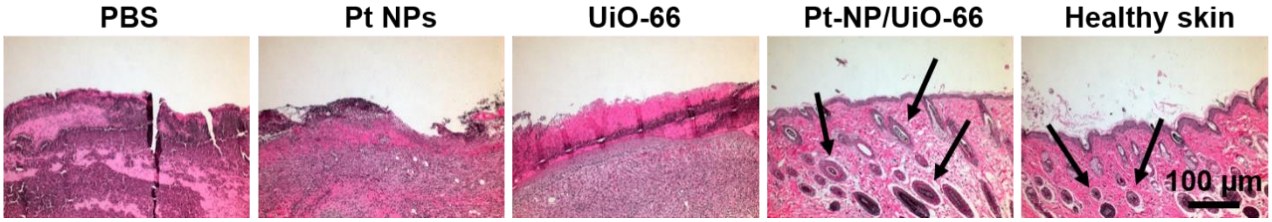

Figure S12. Histological micrographs of infected wound tissue in mice at sacrifice, i.e. 12 days after initiating injection of PBS, Pt nanoparticles, UiO-66 or Pt-NP/UiO-66 MOFs. Tissue of a non-infected, healthy mouse is added for comparison. Tissue was stained with Hematoxylin-Eosin prior to microscopic examination. Arrows point to (restored) hair follicles.

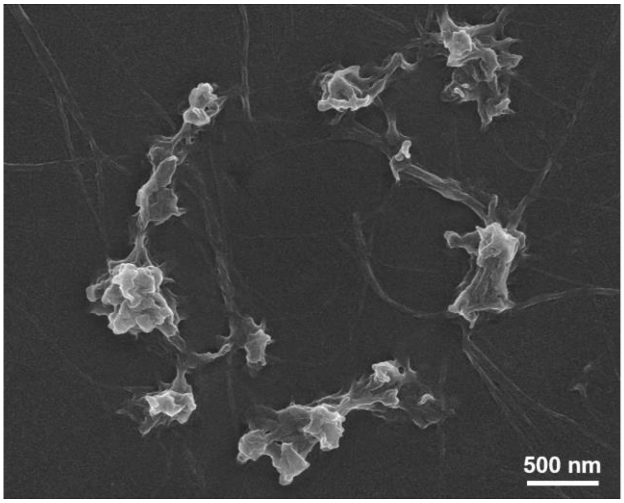

Figure S13. SEM micrograph of bacterial fragments, obtained by exposure to Pt-NP/UiO-66 MOFs for 8 h in PBS at pH 7.

Table S1. Activity levels of mice after different treatment of wound infection. "Low" indicates near complete inactivity with no playful action, "medium" indicates some activity and playfulness but less than before infection and "high" implies a similar activity level as observed prior to entering the study.

Treatment day	PBS	Pt nanoparticles	UiO-66	Pt-NP/UiO-66
0	Low	Low	Low	Low
4	Low	Low	Low	Medium
8	Medium	Medium	Medium	High
12 (sacrifice)	Medium	Medium	Medium	High

Reference

(1) Katz, M. J.; Klet, R. C.; Moon, S. Y.; Mondloch, J. E.; Hupp, J. T.; Farha, O. K. One Step Backward is Two Steps Forward: Enhancing the Hydrolysis Rate of UiO-66 by Decreasing [OH^-]. *ACS Catalysis*, **2015**, *5*, 4637-4642.
